# Metabolic changes associated with polysaccharide utilization reduce susceptibility to some β-lactams in *Bacteroides thetaiotaomicron*

**DOI:** 10.1128/msphere.00103-24

**Published:** 2024-08-07

**Authors:** Rachael Nilson, Swathi Penumutchu, Francesco S. Pagano, Peter Belenky

**Affiliations:** 1 Department of Molecular Microbiology and Immunology, Brown University, Providence, Rhode Island, USA; University of Nebraska Medical Center College of Medicine, Omaha, Nebraska, USA

**Keywords:** bacteroides, pectin, β-lactam, antibiotic tolerance, carbohydrate utilization, anaerobic

## Abstract

Antibiotic therapy alters bacterial abundance and metabolism in the gut microbiome, leading to dysbiosis and opportunistic infections. *Bacteroides thetaiotaomicron* (*Bth*) is both a commensal in the gut and an opportunistic pathogen in other body sites. Past work has shown that *Bth* responds to β-lactam treatment differently depending on the metabolic environment both *in vitro* and *in vivo*. Studies of other bacteria show that an increase in respiratory metabolism independent of growth rate promotes susceptibility to bactericidal antibiotics. We propose that *Bth* enters a protected state linked to an increase in polysaccharide utilization and a decrease in the use of simple sugars. Here, we apply antibiotic susceptibility testing, transcriptomic analysis, and genetic manipulation to characterize this polysaccharide-mediated tolerance (PM tolerance) phenotype. We found that a variety of mono- and disaccharides increased the susceptibility of *Bth* to several different β-lactams compared to polysaccharides. Transcriptomics indicated a metabolic shift from reductive to oxidative branches of the tricarboxylic acid cycle on polysaccharides. Accordingly, supplementation with intermediates of central carbon metabolism had varying effects on PM tolerance. Transcriptional analysis also showed a decrease in the expression of the electron transport chain (ETC) protein NQR and an increase in the ETC protein NUO, when given fiber versus glucose. Deletion of NQR increased *Bth* susceptibility while deletion of NUO and a third ETC protein NDH2 had no effect. This work confirms that carbon source utilization modulates antibiotic susceptibility in *Bth* and that anaerobic respiratory metabolism and the ETC play an essential role.

**IMPORTANCE:**

Antibiotics are indispensable medications that revolutionized modern medicine. However, their effectiveness is challenged by a large array of resistance and tolerance mechanisms. Treatment with antibiotics also disrupts the gut microbiome which can adversely affect health. *Bacteroides* are prevalent in the gut microbiome and yet are frequently involved in anaerobic infections. Thus, understanding how antibiotics affect these bacteria is necessary to implement proper treatment. Recent work has investigated the role of metabolism in antibiotic susceptibility in distantly related bacteria such as *Escherichia coli*. Using antibiotic susceptibility testing, transcriptomics, and genetic manipulation, we demonstrate that polysaccharides reduce β-lactam susceptibility when compared to monosaccharides. This finding underscores the profound impact of metabolic adaptation on the therapeutic efficacy of antibiotics. In the long term, this work indicates that modulation of metabolism could make *Bacteroides* more susceptible during infections or protect them in the context of the microbiome.

## INTRODUCTION

Antibiotic resistance is a global health crisis that puts an immense burden on healthcare systems and the economy. Gram-negative bacteria are particularly challenging to target because many drugs cannot penetrate the outer cell membrane and efflux pumps will expel drugs that reach the periplasm (1). The Gram-negative anaerobe *Bacteroides thetaiotaomicron* (*Bth*) is an interesting model organism for studying antibiotic resistance because it’s a commensal in the human gut microbiome and an opportunistic pathogen in other body sites. The *Bacteroides* genus constitutes a large fraction of the microbiome and is uniquely adapted to the gut environment (2–4). These bacteria benefit the host by providing protection from pathogen colonization, modulating the immune system, and breaking down dietary polysaccharides that would be otherwise undigestible by the host and other resident microbes (5–7). On the other hand, *Bacteroides* are routinely isolated from anaerobic infections, including those located in the brain, blood, heart, oral cavity, skin, soft tissue, bone, and abdomen (2, 8). Treatment of *Bacteroides* infections can be challenging because they carry many resistance genes and Gram-negative bacteria are less permeable to antibiotics. The dual role that *Bacteroides* play in human health and disease creates an impetus to study the response of *Bth* to antibiotics.

In this study, we focus on the sensitivity of *Bth* to β-lactams, which are the most prescribed class of antibiotics (9). Most β-lactams are not effective as monotherapy for *Bacteroides* infections because these bacteria produce β-lactamases. Since *Bacteroides* are typically involved in polymicrobial infections, their secretion of β-lactamases can also protect other infectious bacteria, further complicating treatment (10, 11). While β-lactamase inhibitors have been developed to increase the efficacy of β-lactams, this strategy is not sufficient on its own because some types of enzymes are not inactivated by the currently available inhibitors, exposure to inhibitors can induce hyperproduction of β-lactamases, and mutations can yield resistant enzymes (12, 13). It would be prudent to develop additional methods to improve the use of the many β-lactams that currently exist. This is important not only from the infectious disease perspective, but also when considering the impact that antibiotics have on the gut microbiome.

Antibiotics are associated with gastrointestinal upset, and research shows that the composition of the microbiome can be significantly altered with antibiotic treatment. In general, antibiotics cause a reduction in microbial diversity which gives pathogens an opportunity to colonize the gut (14, 15). Typically, antibiotic treatment decreases the abundance of Bacteroidetes, Firmicutes, and Actinobacteria while increasing Proteobacteria. This altered microbiota composition is termed dysbiosis and is associated with conditions including inflammatory bowel disease, diabetes mellitus, obesity, and colorectal cancer (14). Frequent antibiotic use in early life can be especially damaging because the normal development of the immune system depends on interactions between the host and the microbiome (16). To improve the use of antibiotics to treat infections and avoid unwanted effects on the microbiome, we must understand their mechanism of action.

β-Lactams inhibit penicillin-binding proteins (PBPs), which cross-link peptidoglycan polymers that are required for bacterial cell wall formation. In general, PBP inhibition causes an imbalance between peptidoglycan synthesis and degradation, resulting in cell lysis (9, 17). However, recent studies have found lysis-independent effects of these drugs, suggesting that the impact of β-lactams on the cell is more complex than previously appreciated (18). One such study found that β-lactams induce toxic futile cycling of peptidoglycan synthesis and degradation that depletes cell wall precursors (19). Investigation of the metabolic response of *Escherichia coli* to bactericidal antibiotics revealed elevated levels of cellular respiration, which ultimately contributed to cell death (20–22). It is evident that the inhibition of PBPs by β-lactams leads to an imbalance in anabolic-catabolic processes in the cell (23). Additionally, an increase in ATP independent of growth rate was shown to be positively correlated with antibiotic lethality (24). Thus, β-lactams induce metabolic shifts and the metabolic state of bacteria determines β-lactam susceptibility (25).

Central carbon metabolism in *Bth* has notable differences from *E. coli* due to *Bth’s* adaptation to the gut environment (26). *Bth* is especially adept at degrading dietary fiber, with an extensive repertoire of polysaccharide utilization loci (PULs) (27). These genes encode proteins that form elaborate complexes within the outer membrane to capture, degrade, and import polysaccharides. Once in the periplasm, hydrolases break down the polysaccharide fragments into monosaccharides that are imported into the cytoplasm. The pathways that *Bth* uses to convert these sugars into energy are not fully elucidated. The conversion of hexoses into pyruvate is likely accomplished via the Embden-Meyerhof-Parnas pathway, then pyruvate is processed through a mix of fermentation and anaerobic respiration (28). *Bth* grown in minimal medium (MM) with glucose as the sole carbon source generates mostly acetate, succinate, formate, and propionate, along with some lactate, as metabolic end products (29). *Bth* has genes for both the reductive and oxidative branches of the tricarboxylic acid (TCA) cycle (26, 30). A recent study found that the electron transport chain (ETC) of *B. fragilis* contains functional Na^+^-pumping NADH:quinone oxidoreductase (NQR) and NADH dehydrogenase II (NDH2) that transfer electrons via a menaquinone pool to fumarate reductase (31). The H^+^-pumping NADH:ubiquinone oxidoreductase (NUO) exists in *B. fragilis* but its role in respiration is unclear due to the lack of E, F, and G subunits which contain the NADH binding site. This type of “headless” NUO complex exists in other bacterial species such as *Prevotella copri* (32). Based on genome annotation, *Bth* carries the NQR, NDH2, and NUO genes including subunits E, F, and G.

A large body of work conducted in *E. coli* and other model organisms has demonstrated that antibiotics cause changes in bacterial metabolism and that changing bacterial metabolism externally causes changes in antibiotic susceptibility. We questioned whether a similar interplay between antibiotics and metabolism might exist in other bacteria in the microbiome. Our previous data suggest that the susceptibility of *Bth* to β-lactams is intrinsically sensitive to carbon source *in vitro* and *in vivo* (33). Specifically, growth on polysaccharides appears to have a protective effect compared to growth on glucose. One of the fibers used was pectin, a polysaccharide composed of different structural regions, with 65% being α-1,4 linked D-galacturonic acid residues that form a backbone (34, 35). Other regions include side chains of varying compositions. Pectin is found in the cell wall of many fruits and is extracted commercially to be used as a food stabilizer, a thickener in cosmetics, and a component of tablets in the pharmaceutical industry (35). Since *Bacteroides*, like *Bth*, tend to be beneficial commensals in the GI but are pathogenic in extra-intestinal tissues, identifying a metabolite-driven and diet-delivered modulation for β-lactam susceptibility could protect gut resident bacteria without impacting β-lactam efficacy in other body sites. In this work, we explored the observed polysaccharide-mediated (PM) tolerance phenotype utilizing minimum inhibitory concentration (MIC) assays, growth curves, RNA-seq, and genetic knockouts under different nutrient conditions.

## RESULTS

### Carbon source modulates the susceptibility of *Bth* to β-lactams independent of β-lactamase activity

**Fig 1:**
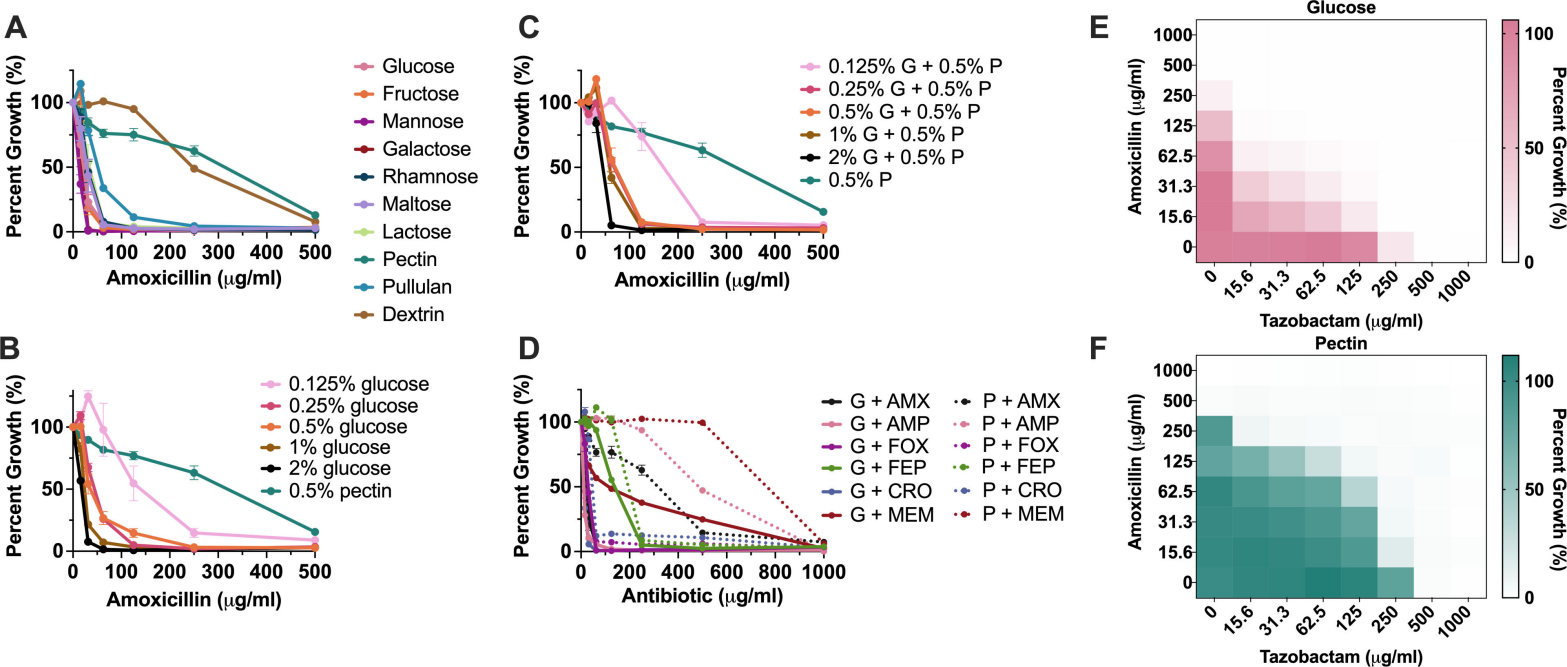
Carbon source modulates the susceptibility of *Bth* to β-lactams independent of β-lactamase activity. (**A–C**) Percent growth plot for AMX treatment of *Bth* grown in MM with different carbon sources (**A**), different concentrations of glucose (**B**), and different concentrations of glucose and pectin (**C**). Data are represented as the average percent growth compared with untreated control cultures ± SEM. G = glucose, P = pectin. (**D**) Percent growth plot for *Bth* grown in MM-glucose or MM-pectin and treated with various β-lactams. Data are represented as the average percent growth compared with untreated control cultures ± SEM. G = glucose, P = pectin, AMX = amoxicillin, AMP = ampicillin, FOX = cefoxitin, FEP = cefepime, CRO = ceftriaxone, MEM = meropenem. (**E and F**) Percent growth plot for AMX plus tazobactam treatment of *Bth* grown in MM-glucose (**E**) or MM-pectin (**F**). Data are represented as the average percent growth compared with untreated control cultures.

In the past, we have shown that the MIC of amoxicillin (AMX) for *Bth* is fourfold higher when *Bth* is grown in MM containing polysaccharides (dextrin, levan, or pectin) compared to glucose as the sole carbon source (33). In this study, we expanded on this initial observation by testing the effects of other monosaccharides, disaccharides, and polysaccharides. We found that the MIC of AMX was 2–16 times higher on polysaccharides compared to mono- and disaccharides (Fig. 1A). We also found that this MIC translated to a difference in kill kinetics between glucose and the polysaccharide dextrin (Fig. S1). We did not find any appreciable differences in the doubling times of *Bth* during growth on these substrates, except for rhamnose and pectin (Fig. S2). Rhamnose is a five-carbon sugar and pentose fermentation has been found to be less energetically efficient than hexose fermentation (36). Growth on pectin was slightly faster than growth on glucose, suggesting that PM tolerance is not dependent on reduced growth rate, as would be suspected based on older concepts of the impact of growth rate on antibiotic susceptibility (Fig. S2) (24). We also found that the terminal optical density (OD) was lower for pectin compared to the other carbon sources and that *Bth* may exhibit biphasic growth on pectin (Fig. S2).

For the remainder of the study, we mostly focused on pectin extracted from citrus peel (purchased from MP Biomedicals) because it had a consistent, robust effect on the MIC in *Bth*. We found that the sensitizing effect of glucose was concentration-dependent and dominant over the protective effect of pectin against AMX (Fig. 1B and C). High concentrations of glucose were associated with higher susceptibly to AMX, in the absence of an observable growth defect (Fig. S3). Adding pectin to MM-glucose led to an intermediate MIC at lower glucose concentrations and had no impact on MIC at higher glucose concentrations (Fig. 1C). A limitation of this data is that we were unable to test higher pectin concentrations due to the low solubility of polysaccharides. We were concerned that because the MM-pectin was cloudy and a small amount of pectin would precipitate, that the pectin could be aggregating AMX and interfering with antibiotic uptake. To check this, we tested the effect of pectin on the MIC of AMX for *E. coli,* which cannot metabolize pectin. We found that the MIC of AMX was the same for *E. coli* grown in LB supplemented with glucose or pectin, indicating that the presence of the polysaccharide itself does not directly inhibit AMX activity (Fig. S4).

Next, we wanted to consider if this phenotype was consistent for other β-lactams and if it was dependent on β-lactamase activity. Interestingly, when testing other β-lactam antibiotics, we found that PM tolerance was also observed with ampicillin and meropenem but not with cefoxitin, cefepime, or ceftriaxone (Fig. 1D). This could indicate that PM tolerance is dependent on the particular mechanism of action of each drug and possibly related to factors such as PBP and β-lactamase specificity (9, 37–40).

The main resistance strategy against β-lactams in Gram-negative bacteria is the production of β-lactamase enzymes (41). Some of these enzymes are constitutively expressed, while others are inducible (42–44). We tested the possibility that pectin induces β-lactamase production by treating *Bth* with both AMX and the β-lactamase inhibitor tazobactam. While the addition of tazobactam lowered the MIC in both conditions, the decrease was far greater in glucose than in pectin, indicating that β-lactamase inhibition has a greater impact in the glucose media (Fig. 1E and F). One explanation of this result is that in the glucose condition, tazobactam has a greater impact because there is relatively more β-lactamase in glucose than in pectin. This would indicate that the protection observed in pectin is likely not driven by β-lactamase production. An alternative explanation for this data is that in the pectin condition, the amount of tazobactam provided is not sufficient to inhibit every available β-lactamase. This would indicate that the protection seen in pectin is largely β-lactamase driven. To discriminate between these two mechanisms, we measured the level of β-lactamase in *Bth* cultures and found that the amount of enzyme was comparable between glucose and pectin growth conditions (Fig. S5A). As an alternative to determine β-lactamase activity in living cells, we incubated *Bth* in pectin or glucose media containing 100 µg/mL of AMX at 30°C. After 4 h of exposure, we treated *E. coli* with this spent media added to LB. Diluted to 50 µg/mL, AMX treated with *Bth* from the glucose condition did not inhibit *E. coli* whereas AMX treated with Bth from the pectin condition showed significant inhibition. This indicates that there is likely more β-lactamase produced in the glucose condition (Fig. S5B). Taken together, these results suggest that the mechanism of PM tolerance is likely β-lactamase-independent, although more work may be warranted in the future.

### Growth on pectin induces a substantially different transcriptional response compared to glucose

To understand what mechanism could be driving PM tolerance, we analyzed the transcriptome of *Bth* in response to AMX in glucose and pectin growth conditions. *Bth* cultures were grown to early log phase (0.1 OD_600_) in either MM-glucose or MM-pectin and treated with three different doses of AMX or vehicle control for 60 min. Samples were collected immediately before treatment (T0) and after 60 min (T60) for each condition. The doses were chosen based on the MICs of AMX for *Bth* grown in MM-glucose or MM-pectin. We selected a dose of 62.5 µg/mL (A62.5) based on the approximate MIC in MM-glucose, a dose of 200 µg/mL (A200) based on a concentration between the MICs in glucose versus pectin, and a dose of 500 µg/mL (A500) based on the MIC in MM-pectin. To confirm relative inhibition, samples were plated for colony-forming units (CFUs). In both the glucose and pectin conditions, the number of CFUs decreased for A200 and A500 at T60, although the observed toxicity was higher in glucose than in pectin (Fig. S6).

**Fig 2:**
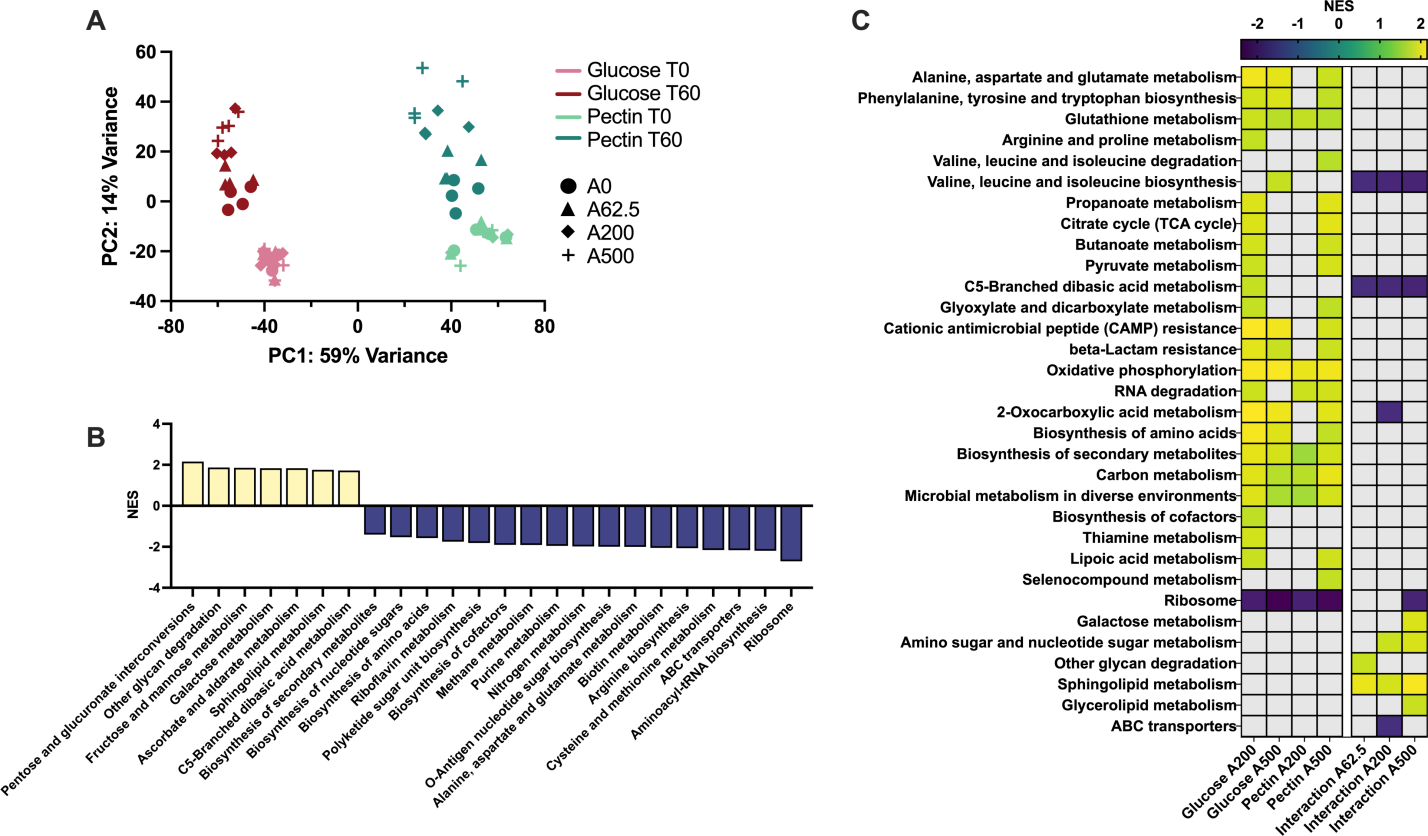
Transcriptional response of *Bth* grown on pectin or glucose with or without AMX treatment. (**A**) PCA plot of variance stabilizing transformation of RNAseq data. (**B and C**) Normalized enrichment scores (NES) for gene set enrichment analysis of KEGG pathways. Benjamini-Hochberg adjusted *P* value < 0.05 and −1 ≤ log_2_fold change ≤ 1. (**B**) Comparison of pectin without AMX versus glucose without AMX. (**C**) Two-way interactions comparing AMX treatment (200 or 500 µg/mL) with no treatment for glucose or pectin separately. Three-way interactions comparing AMX treatment (62.5, 200, or 500 µg/mL) with no treatment for glucose and pectin together, with glucose as the baseline. Gray panels are non-significant. T = time (min), A = amoxicillin dose (μg/mL). For all conditions, *n* = 4.

Principal component analysis (PCA) of RNA-seq data showed that a large portion of the variance among samples was due to carbon source, regardless of AMX treatment (Fig. 2A). Within a given carbon source, samples without AMX clustered separately from samples with AMX, and this was more pronounced with increasing concentrations of AMX. Using DESeq2, we found that 3,271 out of 4,774 genes were differentially expressed when comparing pectin to glucose at T0 without AMX (*P* adj <0.05 and |log_2_fold change| >1). When accounting for changes between T0 and T60 without AMX, the number of differently expressed genes (DEGs) present at each dose of AMX was considerably less than the number of DEGs associated with carbon source alone. Thus, carbon source had a profound effect on the transcriptome of *Bth*, indicating that *Bth* encounters AMX under two vastly different metabolic states while utilizing glucose versus pectin.

To investigate how *Bth* metabolism shifts during growth on pectin versus glucose in more detail, we used clusterProfiler to perform gene set enrichment analysis (GSEA) using both KEGG and GO databases, which identified changes in gene sets associated with each condition. The DEGs found for pectin versus glucose at T0 were positively enriched for gene sets involving carbohydrate degradation and membrane components, while they were negatively enriched for respiration machinery, amino acid metabolism, and the ribosome (Fig. 2B; Fig. S7A). Pectin also activated a number of PULs that have been attributed to pectin degradation in previous studies, such as BT0348-69, BT3043-49, BT3654-58, BT3763-66, and BT4149-77 (45, 46). These data combined with the observation that pectin slightly increases the growth rate support the notion that *Bth* alters not only the direct utilization of mono- vs polysaccharide but also changes how energy and essential molecules are generated under the two conditions.

We also looked at changes in the expression of β-lactam resistance genes and PBPs. We found that the expression of a class A β-lactamase (BT4507) and three putative metallo-β-lactamases (BT1146, BT1410, and BT2578) was higher in pectin compared to glucose, without any AMX. Taken together with the results of the β-lactamase assay (Fig. S5A), this suggests that changes in β-lactamase transcript levels may not translate to changes in β-lactamase activity. The expression of several putative PBP2 genes (BT2501, BT3453, and BT3816) was downregulated in pectin compared to glucose (47). PBP2 is the primary target for most β-lactams in *Bacteroides*, therefore a decrease in its expression could provide protection (48). Taken together with our tazobactam MIC data, these results suggest that there could be multiple causative factors of PM tolerance.

### *Bth* grown on pectin requires a higher dose of amoxicillin to induce a comparable transcriptional response to *Bth* grown on glucose

Next, we looked at the impact of AMX in the context of carbon source by performing GSEA on DEGs produced from two-way interaction analyses between time and AMX treatment. These two-way interactions showed which genes changed between AMX T0 and T60 while accounting for genes that changed during untreated growth between T0 and T60. While changes in a few individual genes were detected for pectin and glucose with A62.5, neither condition showed any enrichment of gene sets (KEGG or GO databases). The β-lactam resistance pathway was upregulated at A200 and A500 for glucose, but only upregulated at A500 for pectin (Fig. 2C). Upregulation in this pathway was mainly due to increases in efflux protein expression. At A200 and A500 for glucose, there was activation of a variety of metabolic pathways, including central carbon metabolism, amino acid metabolism, oxidative phosphorylation, and ATP synthesis coupled proton transport (Fig. 2C; Fig. S7B). These transcriptional responses suggested an overall induction of energy generation, which has been observed after β-lactam administration in other bacteria (20, 22, 23, 49). In contrast, the upregulation of many of these pathways did not happen at A200 for pectin (Fig. 2C; Fig. S7B). *Bth* grown on pectin required a higher dose of AMX to trigger a similar transcriptional antibiotic-induced death response compared to *Bth* grown on glucose. Overlapping pathways between glucose and pectin included activation of the TCA cycle, biosynthesis of amino acids, pyruvate metabolism, and ATP synthesis coupled proton transport (Fig. 2C; Fig. S57B). However, the transcriptional response at A500 for pectin was not identical to the response at A200 for glucose. Taken together, these data suggested that the energy-demanding pathways upregulated in both glucose and pectin with AMX characterized a stereotypical response to β-lactams.

Despite the significant overlap between the genes affected by AMX in the two different carbon sources, a considerable number were unique to glucose or pectin. Amoxicillin significantly altered expression of the following gene sets during growth on glucose but not pectin: arginine and proline metabolism, valine, leucine and isoleucine biosynthesis, C5-branched dibasic acid metabolism, biosynthesis of cofactors, thiamine metabolism, four iron, four sulfur cluster binding, transmembrane transport, transporter activity, DNA binding, cytoplasm, and ligase activity (Fig. 2C; Fig. S7B). On the other hand, AMX led to enrichment of the following gene sets unique to pectin: valine, leucine, isoleucine degradation, selenocompound metabolism, receptor activity, DNA-templated regulation of transcription, polygalacturonase activity, β-galactosidase activity, and β-galactosidase complex (Fig. 2C; Fig. S7B).

Some gene sets were modulated at A200 for glucose, but these changes were absent at the higher dose. This observation likely has to do with the higher toxicity and bacterial death at A500, erasing drug and carbon source-specific changes. For example, the TCA cycle and pyruvate metabolism were upregulated at A200 for glucose but had no significant change at A500. On the contrary, this upregulation was not observed for pectin until the A500 dose.

GSEA performed on DEGs produced from an analysis of the three-way interaction between time, carbon source, and AMX treatment allowed us to test whether the effect of a 60-min treatment with AMX was significantly different across carbon source. Two-way interactions looked at the response in glucose to AMX separately from the response in pectin to AMX and while we can compare these responses, we cannot determine which differences in response are due specifically to carbon source. This is accomplished with three-way interaction analyses, providing additional information that was not captured by the two-way interactions. Gene sets enriched in the three-way interactions have the strongest association with the PM tolerance phenotype because all three factors (time, AMX treatment, and carbon source) are necessary to explain the enrichment.

Gene sets that were enriched in both the two-way and three-way interactions for the same AMX dose indicated the magnitude of a change induced by AMX was different based on carbon source. For example, ATP-coupled proton transport was upregulated for pectin at A500 but downregulated for the three-way interaction at A500 (Fig. S7B). This meant that although genes involved in ATP-coupled proton transport were upregulated in pectin treated with 500 µg/mL AMX, the upregulation was not as strong as it was in glucose.

Gene sets that were enriched for a three-way interaction, but not a two-way interaction indicated processes not significantly changed by AMX treatment within a given carbon source but for which carbon source did significantly impact the response to AMX. An example of this was potassium ion transport, which was downregulated in pectin compared to glucose treated with AMX, only in the three-way interaction (Fig. S7B). Galactose metabolism, amino sugar metabolism, nucleotide sugar metabolism, sphingolipid metabolism, glycerolipid metabolism, and other glycan degradation were upregulated for some of the three-way interactions but were not significantly changed for any two-way interaction (Fig. 2C and S7B). This indicated that these key pathways likely changed in opposite directions in the two different carbon sources.

### Glycolytic and TCA cycle metabolites alter the effect of carbon source on *Bth* susceptibility

**Fig 3:**
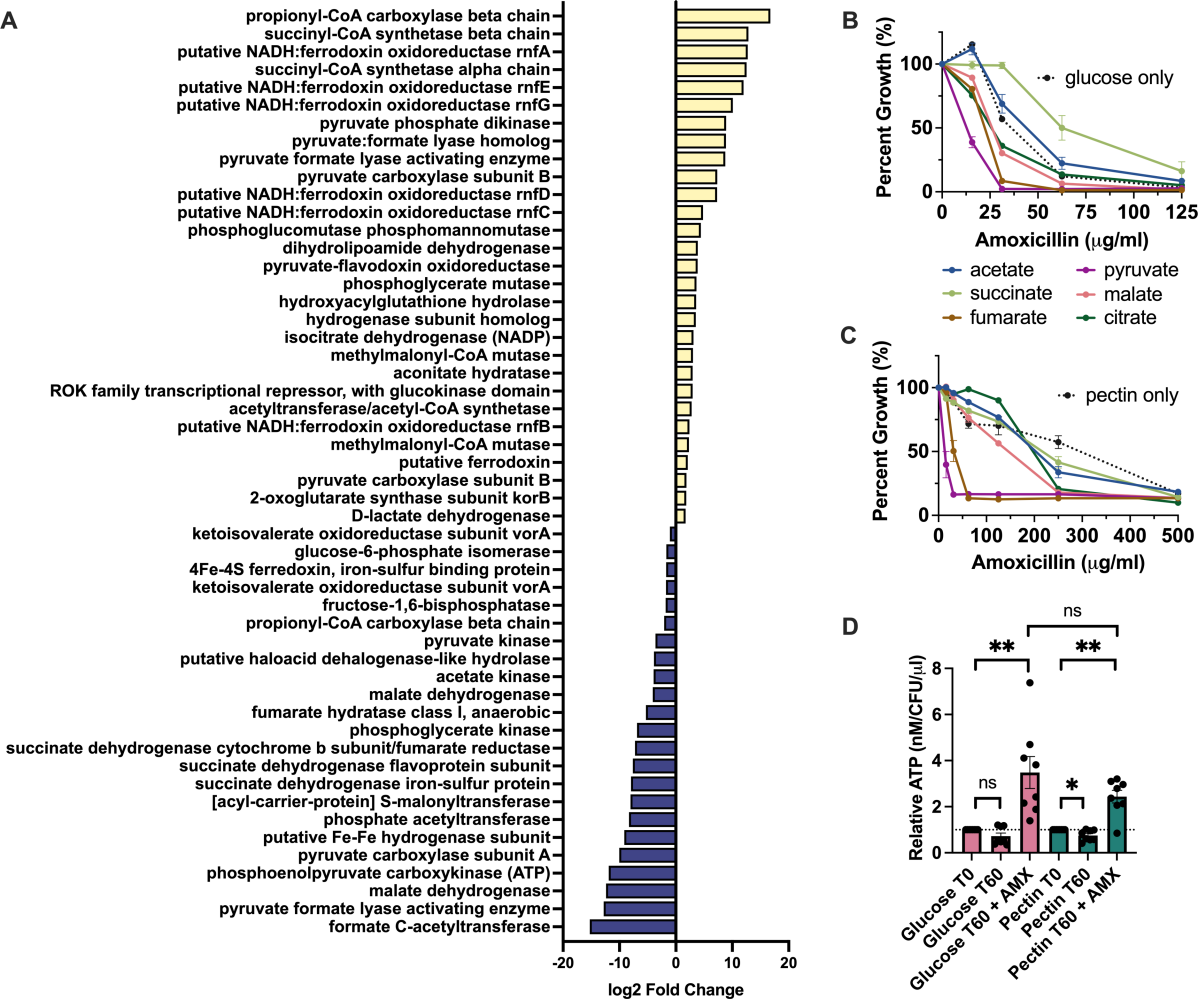
Glycolytic and TCA cycle metabolites alter the effect of carbon source on *Bth* susceptibility and ATP levels are consistent between carbon sources. (**A**) Log_2_ fold change for genes involved in glycolysis and the TCA cycle, comparing pectin T0 A0 to glucose T0 A0. Benjamini-Hochberg adjusted *P* value < 0.05. (**B and C**) Percent growth plot for AMX treatment of *Bth* grown in MM-glucose (**B**) or MM-pectin (**C**) supplemented with metabolites. Data are represented as the average percent growth compared with untreated control cultures ± SEM. (**D**) Relative ATP concentrations for *Bth* grown in MM-glucose or MM-pectin and treated with AMX. ATP concentrations were normalized to cell number. Data are represented as mean ± SEM. *P < 0.05, **P < 0.01; paired *t* test. T = time (min), A = amoxicillin dose (μg/mL).

Many studies support the hypothesis that antibiotic lethality is caused in part by changes in TCA cycle metabolism which lead to the generation of reactive species (20, 21, 23–25, 49–51). Our transcriptomics data showed differences in enzymes involved in glycolysis, fermentation, and the TCA cycle in pectin compared to glucose. Without AMX, some enzymes were upregulated while others were downregulated (Fig. 3A). Although glycolytic and TCA pathways are not fully defined in *Bth*, comparison with other bacteria suggested that in pectin, *Bth* upregulated the oxidative branch of the TCA cycle to form succinyl CoA and produced short-chain fatty acids (SCFAs) as metabolic byproducts of fermentation. This coincided with downregulation of enzymes that would lead to anaerobic respiration with fumarate as a terminal electron acceptor. For example, malate dehydrogenase, fumarate hydratase, and fumarate reductase were downregulated in pectin, while citrate aconitase, isocitrate dehydrogenase, 2-oxoglutarate synthase, and succinyl-CoA synthetase were upregulated. The upregulation of pyruvate:flavodoxin oxidoreductase (PFOR), pyruvate-formate lyase (PFL), a hydrogenase, and ferrodoxin:NADH oxidoreductase (Rnf) along with the downregulation of phosphoenolpyruvate (PEP) carboxykinase in pectin suggested that a large portion of pyruvate was likely processed into acetyl-CoA rather than oxaloacetate. This would be accompanied by the production of formate, CO_2_, and H_2_ as byproducts.

Since our transcriptomics data indicated that antibiotic-induced changes in metabolic pathways depend on carbon source, we tested whether the addition of certain metabolites during AMX treatment would affect PM tolerance. Pyruvate feeds into both branches of the TCA cycle and the formation of SCFAs; therefore, it serves as a starting point for many pathways that were modulated in our data. Fumarate is the terminal electron acceptor for anaerobic respiration and the activity of fumarate reductase is important for energy generation in *Bacteroides* (52). Malate and citrate are intermediates of the reductive and oxidative TCA branches, respectively. Acetate and succinate are major secreted metabolites in *Bth* and an excess of either could cause feedback inhibition (29). Adding pyruvate or fumarate to the glucose medium decreased the MIC (Fig. 3B). Both acetate and succinate slightly increased the MIC, while malate or citrate had no effect on glucose. Interestingly, adding pyruvate or fumarate to the pectin medium decreased the MIC of AMX, eliminating the PM tolerance phenotype despite no change in the growth rate (Fig. 3C; Fig. S8). The addition of malate or citrate to pectin also lowered the MIC but the effect was not as robust as the effect of pyruvate or fumarate. On the other hand, neither acetate nor succinate influenced the MIC for pectin.

Previous studies have indicated that the toxicity of β-lactams may be in part due to the increased metabolic burden of peptidoglycan synthesis, which includes an increase in ATP demand (19, 23, 53–55). To determine if this was the case in *Bth* treated with AMX, we measured ATP levels in cells treated with 200 µg/mL AMX for sixty minutes. We found that under both pectin and glucose conditions, AMX induced a significant boost in cellular ATP concentrations (Fig. 3D). This indicated that while *Bth* utilized different metabolic pathways with different carbon sources (Fig. 3A), the requirement for ATP production remained consistent.

### *Bth* utilizes different NADH:quinone oxidoreductases depending on carbon source

**Fig 4:**
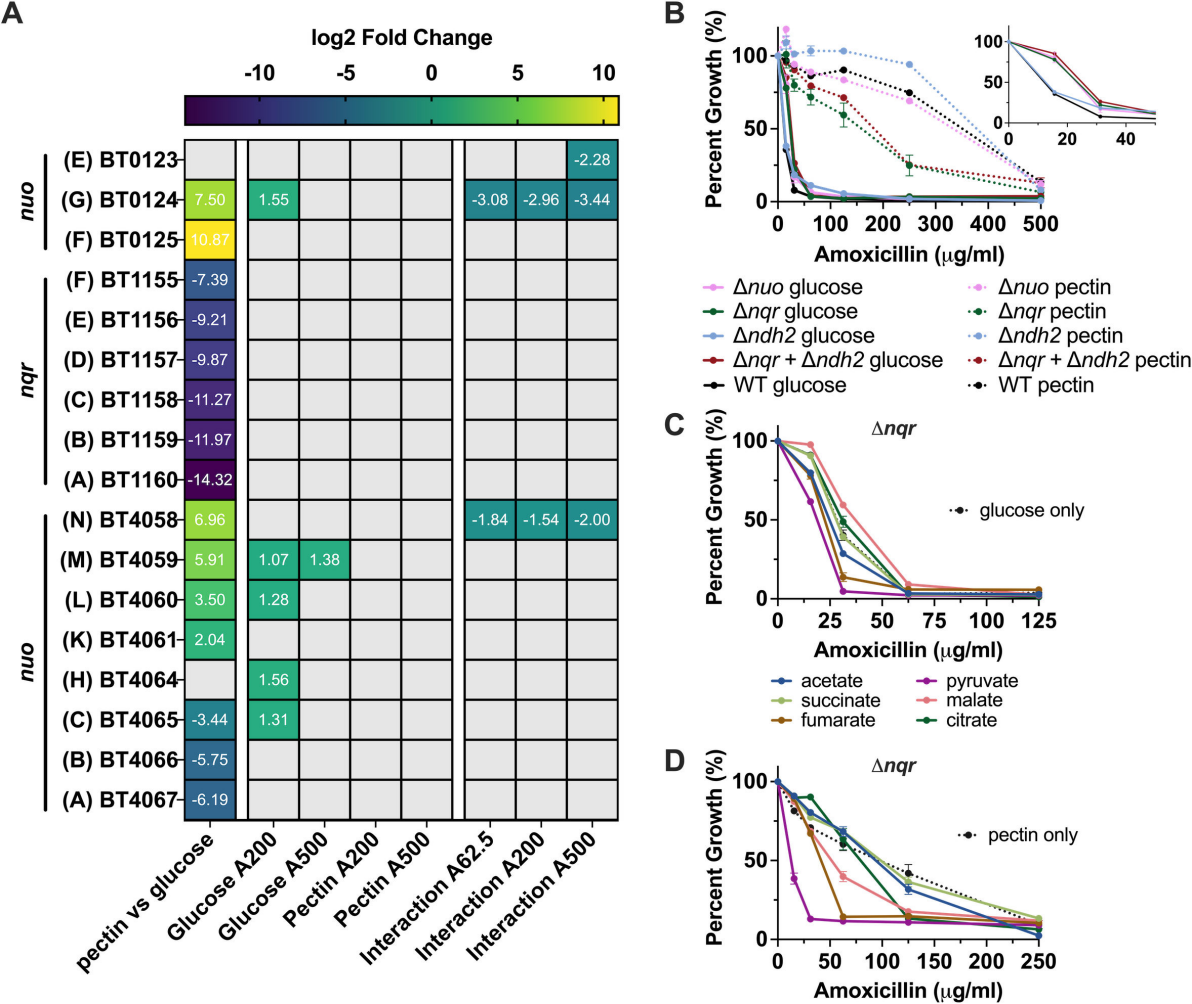
*Bth* utilizes different NADH:quinone oxidoreductases depending on carbon source. (**A**) Log_2_ fold change for *nuo* and *nqr* operons, with letters designating subunits. Comparisons are pectin without AMX versus glucose without AMX, two-way interactions comparing AMX treatment (200 or 500 µg/mL) with no treatment for glucose or pectin separately, and three-way interactions comparing AMX treatment (62.5, 200, or 500 µg/mL) with no treatment for glucose and pectin together, with glucose as the baseline. Benjamini-Hochberg adjusted *P* value <0.05. Gray panels are non-significant. A = amoxicillin dose (μg/mL). (**B**) Percent growth plot for AMX treatment of *Bth* knockouts grown in MM-glucose or MM-pectin. Data are represented as the average percent growth compared with untreated control cultures ± SEM. (**C and D**) Percent growth plot for AMX treatment of *Bth* Δ*nqr* grown in MM-glucose (**C**) or MM-pectin (**D**) supplemented with metabolites. Data are represented as the average percent growth compared with untreated control cultures ± SEM.

We wanted to explore ETC gene expression because our transcriptomics data had shown an increase in pathways and enzymes involved in anaerobic respiration. There was differential expression of NQR and NUO subunits when comparing pectin vs glucose without AMX (Fig. 4A). All subunits (A–F) of NQR were downregulated in pectin versus glucose. In contrast, subunits A-CD of NUO were downregulated, subunits F, G, K–M were upregulated, and subunits E, H–J had no significant difference. We found no significant difference in the expression of NDH2 in pectin vs glucose. There were only small changes in the expression of a few subunits with AMX treatment; therefore, the baseline difference in ETC genes seemed to be more relevant to PM tolerance than the effect of AMX (Fig. 4A).

Based on these data and the effects of TCA cycle intermediates on MIC, we generated in-frame deletions of the *nqr* operon, the *nuo* operon (excluding subunits E, F, and G), and the *ndh2* gene. The Δ*nqr* and Δ*nqr*Δ*ndh2* mutants had shifted MICs and increased growth inhibition under intermediate AMX concentrations with pectin, despite no change in the doubling time of Δ*nqr* and an increase in the doubling time for Δ*nqr*Δ*ndh2* (Fig. 4B ; Fig. S9). The Δ*nuo*, Δ*nqr*, and Δ*nqr*Δ*ndh2* mutants had decreased inhibition under low AMX concentrations with glucose (Fig. 4B, inset). As was seen with pectin, only the Δ*nqr*Δ*ndh2* mutant showed a change in growth rate, at approximately 1.5 times that of the WT grown in glucose (Fig. S9). The inconsistency between changes in growth rate and changes in susceptibility may support the notion that when growth rate is decoupled from metabolic activity, it does not necessarily have an impact on antibiotic susceptibility (24). Overall, the change in the pectin MIC curve observed in the Δ*nqr* and Δ*nqr*Δ*ndh2* mutants indicated that the differential utilization of these complexes might partially explain the observed PM tolerance phenotype.

To further explore the Δ*nqr* mutant, we determined the MIC of AMX in glucose and pectin with the addition of the same metabolites that had been tested in the WT. Adding pyruvate or fumarate to the glucose medium decreased the MIC of AMX, while malate and citrate had no effect, matching results in the WT (Fig. 4C). Unlike in the WT, acetate and succinate had no effect on the MIC in glucose. The lack of protective effects of succinate and acetate in the Δ*nqr* mutant with glucose suggested that these metabolites may not play as significant of a role in metabolism as they do in the WT. The effects of the metabolites in pectin on the MIC for the Δ*nqr* mutant were the same as the effects in the WT (Fig. 4D). Adding pyruvate, fumarate, malate, or citrate to the pectin medium decreased the MIC while neither acetate nor succinate had an effect. Most of the metabolites produced the same growth rate in Δ*nqr* as they did in the WT, except that the doubling time of Δ*nqr* in glucose plus acetate was increased and the doubling time of Δ*nqr* in pectin plus pyruvate was increased (Fig. S10).

## DISCUSSION

Metabolism and antibiotic efficacy are strongly linked in model organisms such as *E. coli* (20, 22, 23, 49, 56). However, due to the wide range of metabolic capabilities across different types of bacteria, it is important to investigate the effects of antibiotics in diverse host-associated microbes. We found that the ability of some β-lactams to inhibit *Bth* growth is dependent on carbon source *in vitro*. While we have referred to this phenotype as PM tolerance, it is also important to highlight the sensitizing effect of glucose. Due to the apparent dominant effect of glucose (Fig. 1C), it may be that the clinical relevance of this work lies in the mitigation of glucose levels, as opposed to the use of pectin as a potential prebiotic.

There are many factors that determine the spectrum of activity for a given β-lactam, including its ability to penetrate the cell, its affinity for PBPs, and its resistance to degradation by β-lactamases. AMX and ampicillin (AMP) are relatively narrow spectrum and very similar structurally; therefore, it is not unexpected that if one shows the PM tolerance phenotype, the other one will as well. Cefoxitin, cefepime, and ceftriaxone are broad-spectrum cephalosporins and part of a separate class of β-lactams compared to AMX and AMP. The structural differences between penicillins and cephalosporins may be the reason why the cephalosporins that we tested did not induce PM tolerance. Interestingly, meropenem had a different inhibition profile in glucose versus pectin even though it is not structurally similar to AMX or AMP. Meropenem is a broad-spectrum carbapenem resistant to most β-lactamases and as a result is used clinically to treat serious anaerobic infections, especially those involving *Bacteroides* (8, 57). Despite having the same primary target, it is clear that different β-lactams have nuanced effects on the cell and further investigation is needed to understand why PM tolerance is not consistent across this class of antibiotics.

An additional implication evident from the checkerboard assay is that tazobactam is more effective in conditions where the bacteria are utilizing glucose rather than pectin. During a typical non-GI infection, *Bth* is unlikely to utilize large dietary polysaccharides and would thus be more susceptible to combination therapy with a β-lactam/β-lactamase inhibitor, whereas *Bth* in its commensal niche would still be protected. This is an important therapeutic implication that should be explored further. A limitation to this study is that the concentrations of drug used may not be relevant in a clinical setting. According to the Clinical and Laboratory Standards Institute, the MIC breakpoint for resistance in *Bacteroides* is ≥16/8 µg/mL for amoxicillin-clavulanate and ≥16 µg/mL for meropenem (58). These tests are typically conducted in Brucella broth supplemented with hemin, vitamin K_1_, and lysed horse blood and these results are likely not transferable to our fully defined minimal media conditions (59). In general, oral administration of amoxicillin results in peak serum levels between 5 and 10 µg/mL, depending on the dose; however, tissue and GI levels could vary dramatically (60, 61). While pectin conditions required much higher concentrations of AMX to inhibit growth, we did find small differences in susceptibility under glucose conditions with some of the mutants (Fig. 4B). These differences occurred at AMX concentrations that are close to therapeutic levels.

Our finding that *Bth* had a very similar transcriptional response to AMX in glucose as it did to a higher dose of AMX in pectin suggests that the antibiotic mechanism of action is the same in both carbon sources. It is likely that the initial metabolic state of *Bth*, which is determined in part by carbon source, affects susceptibility. Earlier, we found the requirement for ATP production remained consistent during antibiotic exposure despite the carbon source. Our transcriptomics data showed that *Bth* uses different metabolic pathways to generate ATP in glucose vs pectin. Therefore, *Bth* appears to rely more on the ETC for ATP generation in the glucose condition compared to pectin. Alternatively, under these conditions, it appears that pectin is processed mainly through glycolysis to yield SCFAs as a byproduct and through the oxidative branch of the TCA cycle. Although it is likely that both carbon sources are processed through a combination of anaerobic respiration and fermentation, it is possible that the ratio of utilization of these pathways may be different between glucose and pectin.

Much of what is known about the role of the ETC in anaerobic respiration has been learned from *E. coli*. Recent studies have shown that *B. fragilis* utilizes NQR and NDH2 during anaerobic respiration (31, 62). The role of NQR, NDH2, and NUO in *Bth* has yet to be explored; however, our data suggest that NQR is not utilized as much in pectin compared to glucose conditions. The NUO complex in *B. fragilis* lacks the E-F-G subunits, which bind NADH, and Ito et al. attribute this as the reason why it does not display NADH reductase activity in this organism. The *Bth* genome does contain homologs of the E-F-G subunits; however, they are not encoded within the same operon as the rest of the subunits, as they are in *E. coli*. The effect of this on the function of NUO in *Bth* is unknown but it is likely that the complex does not have the same conformation that it does in *E. coli*. It was shown that expression of only the E-F-G subunits in *E. coli* can form an active complex in the cytoplasm; therefore, NUO may still participate in the *Bth* ETC (63). Since we found a downregulation in *nqr* genes with pectin, it is possible that *Bth* utilizes NQR to regenerate NAD+ when metabolizing glucose and NUO to regenerate NAD+ when metabolizing pectin. The generation of ATP using the ETC requires the activity of fumarate reductase; therefore, the decrease in fumarate reductase expression in pectin suggests that ATP is generated via other non-respiratory pathways such as substrate-level phosphorylation.

Since *Bth* appears to rely mainly on NQR to generate ATP under the glucose condition and NUO to generate ATP under the pectin condition, we initially hypothesized that the Δ*nqr* mutant would be protected from AMX with glucose. However, we found that the inhibitory effect of AMX on the Δ*nqr* mutant was the same for glucose but stronger for pectin compared to WT. A possible explanation for this is that without NQR, *Bth* growing on pectin cannot meet its ATP demands through solely substrate-level phosphorylation and use of the NUO complex. Therefore, the Δ*nqr* mutant growing on pectin must process pectin through mainly anaerobic respiration using NUO and potentially other ETC proteins that altogether potentiate AMX treatment.

There are limitations to this interpretation because most *Bth* gene annotations have not been experimentally validated and many are predicted based on studies in *E. coli*, which is only distantly related to *Bth* (64). Additionally, changes in gene transcription do not necessarily correlate to changes in protein levels or protein activity (65, 66). Future investigations using qPCR, enzyme assays, and metabolomics would help improve our understanding of PM tolerance. In particular, the differential expression of PBP2 may provide an important mechanism driving this tolerance. This could be investigated using the fluorescent penicillin analogue, bocillin-FL, to measure how much β-lactam the cells bind and to determine which PBPs are targeted in PM tolerance (47, 67). It is possible that changes in the composition of the cell membrane that are necessary for pectin utilization include changes in PBP expression or activity. This would have an incidental effect on β-lactam susceptibility. Deciphering the mechanism behind this phenotype could help identify future therapeutic targets to combat multidrug resistance. The ability to possibly modulate metabolism to alter susceptibility is also relevant to protecting *Bth* in the microbiome. Dietary interventions, such as instructing patients to increase their fiber intake while taking antibiotics, are one avenue of exploration. Altogether, this work provides a novel investigation of carbon source-mediated tolerance in an important host-associated microbe.

## MATERIALS AND METHODS

### Bacterial strains and growth conditions

*Bth* (VPI-5482) was purchased from the American Type Culture Collection (ATCC; Manassas, VA, USA). All strains of *Bacteroides* were cultured in modified Gifu Anaerobic Media (mGAM; HyServe) or *Bacteroides* MM (68) containing 0.5% (wt/vol) carbon source. To prepare MM, first a 10× salt solution with pH 7.2 was made containing 1 M KH_3_PO_4_, 150 mM NaCl, and 85 mM (NH_4_)_2_SO_4_. Next, the 10× salt solution was combined with supplements and filtered through a 0.2 µm filter to produce 2× MM without carbon source. Finally, this solution was combined with a 1% carbon source solution to yield MM with the following composition: 100 mM KH_3_PO_4_, 15 mM NaCl, 8.5 mM (NH_4_)_2_SO_4,_ 4 mM l-cysteine, 1.9 μM hemin, 200 μM l-histidine, 100 μM MgCl_2_, 1.4 μM FeSO_4_·7H_2_O, 72 μM CaCl_2_, 1 μg/mL vitamin K_3_, 5 ng/mL vitamin B_12_, 1 mg/L resazurin, and 0.5% (wt/vol) carbon source. Supplement stock solutions were stored at 4°C for <6 months, except for the l-histidine and FeSO_4_·7H_2_O stock solutions which were made fresh. *Bacteroides* were grown at 37°C in either a Coy anaerobic chamber (Coy Labs, Grass Lake, MI, USA) under an atmosphere of 2.5% H_2_, 5% CO_2_, 92.5% N_2_ or a BactronEZ anaerobic chamber (Sheldon Manufacturing, Cornelius, OR, USA) under an atmosphere of 5% H_2_, 5% CO_2_, 90% N_2_. *E. coli* strain MG1655 (ATCC# 700926) was cultured in Luria-Burtani (LB) and grown aerobically at 37°C. For MIC determination, *E. coli* was grown in LB supplemented with 0.5% (wt/vol) glucose or 0.5% (wt/vol) pectin. *E. coli* S17 λ pir was used for conjugation of deletion plasmids into *Bth* (68). Bacterial strains can be found in Table S1.

Stock solutions of monosaccharides and disaccharides were prepared by dissolving 1% (wt/vol) of the carbon source in water and sterilizing by filtration through a 0.2 μm filter. Stock solutions of polysaccharides were prepared by adding 1% (wt/vol) of the carbon source to at least 500 mL of water, autoclaving for a sterilization time of 20 min at 121°C, and subsequently storing at 4°C (69). We found that smaller volumes of polysaccharide solutions or longer sterilization times were associated with variable MICs, which was likely a result of polysaccharide degradation into monosaccharides (70). Pectin (MP Biomedicals) and pullulan (Alfa Aesar) were purchased through Thermo Fisher and dextrin was purchased through Sigma-Aldrich.

Metabolites were added to MM by preparing the 10× salt solution with 100 mM of sodium pyruvate, disodium fumarate, disodium succinate hexahydrate, sodium citrate dihydrate, disodium dl-malate hydrate, or sodium acetate trihydrate. This yielded a final concentration of 10 mM of metabolite in MM after the salt solution was combined with the supplements and carbon source as described above.

### MIC determination

MICs were determined using the broth dilution method (71). In short, overnight cultures of *Bacteroides* grown in mGAM were diluted 100-fold into MM with different carbon sources. Overnight cultures of *E. coli* grown in LB were diluted 10,000-fold in LB containing different carbon sources. Amoxicillin, ampicillin sodium salt, cefoxitin sodium, ceftriaxone sodium, cefepime hydrochloride monohydrate, or meropenem trihydrate (purchased through Thermo Fisher or Sigma-Aldrich) were added at varying concentrations to cell culture media and serially diluted twofold. Cells were then incubated anaerobically (*Bacteroides*) or aerobically (*E. coli*) at 37°C for ~20 h, after which OD_600_ readings were taken on a SpectraMax M3 microplate reader (Molecular Devices LLC, San Jose, CA, USA). Data represent the average of at least three biological replicates.

Antibiotic stock solutions were prepared fresh by dissolving amoxicillin and meropenem in DMSO and dissolving the other antibiotics in water and filtering (0.2 μm). We found DMSO had a slight inhibitory effect on cell growth at concentrations above 2%.

### Checkerboard assay

A checkerboard microdilution assay was used to determine the interaction between amoxicillin and tazobactam. First, amoxicillin was dissolved in a 1:1 solution of DMSO and water and then serially diluted twofold in MM-glucose or MM-pectin in a 96-well plate. Tazobactam sodium was dissolved in water and then serially diluted twofold in MM-glucose or MM-pectin in a separate 96-well plate. The amoxicillin and tazobactam dilutions were transferred to a 96-well plate containing *Bth* diluted 1:100 in MM-glucose or MM-pectin. The resulting assay plate contained amoxicillin diluted along the ordinate and tazobactam diluted along the abscissa. Final concentrations of amoxicillin were: 1,000, 500, 250, 125, 62.5, 31.25, and 15.63 μg/mL. Final concentrations of tazobactam were: 10, 5, 2.5, 1.25, 0.63, 0.31, and 0.16 μg/mL. Cells were then incubated anaerobically at 37°C for ~20 h, after which OD_600_ readings were taken on a SpectraMax M3 microplate reader (Molecular Devices LLC, San Jose, CA, USA). Data represent the average of five biological replicates.

### Amoxicillin treatment of log phase *Bth*

Overnight *Bth* cultures grown in mGAM were pelleted and resuspended in MM-glucose or MM-pectin. Resuspended cultures were diluted to an OD_600_ of 0.05 and grown to 0.1. Each culture was split into four aliquots and T0 samples were taken for RNA extraction and CFU plating. Next, amoxicillin dissolved in DMSO was added to three of the four aliquots to yield 500, 200, and 62.5 μg/mL, while DMSO was added to the fourth aliquot. After 60 min of anaerobic incubation at 37°C, T60 samples were taken for RNA extraction and CFU plating. For the RNA extraction samples, 1.5 mL of culture was pelleted at 8,000 × g for 2 min, resuspended in 1 mL RNAlater, and stored at 4°C until extraction (<1 month at 4°C). For CFU determination, the cells were serially diluted 10-fold in PBS and plated on mGAM. Plates were incubated at 37°C until colonies were visible for counting (~20 h).

### RNA extraction and quantification

Samples were pelleted, washed once in PBS, then resuspended in lysis buffer from the Quick-RNA Fungal/Bacterial Miniprep Kit (Zymo Research, Irvine, CA, USA). Total RNA was extracted from cells using this kit as per the manufacturer’s instructions, including DNase I treatment. RNA was eluted in nuclease-free water and stored at −80°C until further use. Total RNA was quantified using the RNA-HS kit on a Qubit 3.0 Fluorometer (Thermo Fisher Scientific, Waltham, MA, USA). RNA integrity was assessed using an Agilent 2100 Bioanalyzer (Agilent, Santa Clara, CA, USA).

### RNA-seq library preparation

Libraries were prepared from total RNA (~500 ng) using the NEBNext rRNA Depletion Kit (Bacteria) and the NEBNext Ultra II Directional RNA Library Prep Kit (New England Biolabs, Ipswich, MA, USA) following the manufacturer’s instructions for intact RNA. Amplification was done using NEBNext Multiplex Oligos (96 unique dual index primer pairs). Libraries had an average fragment size of 280–360 bp, assessed using an Agilent Fragment Analyzer (Agilent, Santa Clara, CA, USA). The library of pooled samples was sequenced on an Illumina NovaSeq 6000 using paired-end, 150 bp reads. We obtained an average of 7,924,142 (±3,068,105) raw reads per sample.

### Preprocessing of raw sequencing reads

Transcriptomic reads were trimmed and decontaminated using KneadData (version 0.6.1) (72). Trimming was done using Trimmomatic (version 0.39) with a SLIDINGWINDOW of 4:20 (73). Contaminating reads from the SILVA 128 database containing LSU and SSU ribosomal RNA sequences were removed using Bowtie2 (version 2.3.5.1) (74, 75).

### RNA-seq analysis

Processed reads were aligned to NCBI GenBank Assembly GCF_000011065.1 using Bowtie2 (version 2.3.4.5.1) (75). Aligned reads were counted using the featureCounts command in the subread program (version 1.6.2) (76). Next, counts were input into DESeq2 (version 1.40.2) for differential expression analysis with multiple hypothesis testing correction using the Benjamini-Hochberg method (77, 78). Finally, GSEA was performed using clusterProfiler (version 4.8.2) with default settings, except minGSSize was set to 5 (79). DESeq2 and clusterProfiler analyses were run in R version 4.3.1.

### ATP measurement

Overnight *Bth* cultures grown in mGAM were pelleted and resuspended in MM-glucose or MM-pectin. Resuspended cultures were diluted to an OD_600_ of 0.05 and grown to 0.1. Each culture was split into two aliquots and T0 samples were taken for ATP measurement. Next, amoxicillin dissolved in DMSO was added to one aliquot to yield 200 µg/mL, while DMSO was added to the other aliquot. After 60 min of incubation at 37°C, T60 samples were taken for ATP measurement. ATP was measured with the BacTiter-Glo Microbial Cell Viability Assay (Promega, Madison, WI, USA), as per the manufacturer’s instructions. The BacTiter-Glo reagent was equilibrated inside the anaerobic chamber for at least 1 h and the assay plate was set up inside the chamber. Briefly, each cell culture sample was diluted 1:100 in MM-glucose or MM-pectin media, and a 100 µL aliquot of this dilution was mixed with 100 μL BacTiter-Glo reagent in an opaque 96-well plate. Samples were mixed with a multichannel pipet and incubated at room temperature for 5 min. Luminescence was measured using the default settings of the CellTiter-Glo protocol on a SpectraMax M3 microplate reader (Molecular Devices LLC, San Jose, CA, USA). Standard curves for the MM-glucose media and the MM-pectin media were generated to calculate ATP concentrations. For CFU determination, the cells were serially diluted 10-fold in PBS and plated on mGAM. Plates were incubated at 37°C until colonies were visible for counting (~20 h). ATP concentrations were normalized to CFU count.

### Growth rate determination

Overnight cultures of *Bth* grown in mGAM were diluted 1:100 into minimal media containing 0.5% (wt/vol) of carbon source. Cells were incubated at 37°C under anaerobic conditions and growth was monitored by a stratus kinetic microplate reader (Cerillo, Charlottesville, VA, USA) shaking at ~200 rpm on an IKA MS 3 basic orbital shaker (IKA, Wilmington, NC, USA). OD_600_ readings were recorded at 30-min intervals. To determine the doubling time in each strain and condition, a logarithmic segment of the growth curve was fitted to an exponential growth function using default settings in Prism (version 10). Doubling times are an average of at least two biological replicates. The OD_600_ data for each replicate were graphed using Prism software. All growth data can be found in Tables S3 to S7.

### Strain construction

In-frame genetic deletions were performed by counter-selectable allelic exchange as previously described (68). The *Bth* single deletion mutants have the following genes deleted: Δ*nqr* (BT1155–1160), Δ*ndh2* (BT0387), and Δ*nuo* (BT4058–4067). The Δ*nuo* mutant still possesses the EFG subunits (BT0123–0125), which are not located within the same operon as the rest of the Δ*nuo* subunits. The *Bth* double deletion mutant Δ*nqr*Δ*ndh2* was created by deleting *ndh2* in the Δ*nqr* strain. Strains and plasmids can be found in Table S1, and primers can be found in Table S2.

## Data Availability

RNA-seq data have been deposited in NCBI’s Gene Expression Omnibus (80) and are accessible through GEO Series accession number GSE251676.

## SUPPLEMENTAL MATERIAL

Supplemental figures, tables, methods msphere.00103-24-s0001.docx Supplemental figures, Tables S1 and S2, and methods.

Table S3 msphere.00103-24-s0002.xlsx WT carbon source doubling times.

Table S4 msphere.00103-24-s0003.xlsx WT glucose concentration doubling times.

Table S5 msphere.00103-24-s0004.xlsx WT metabolite doubling times.

Table S6 msphere.00103-24-s0005.xlsx KO doubling times.

Table S7 msphere.00103-24-s0006.xlsx Δ*nqr* metabolite doubling times.

ASM does not own the copyrights to Supplemental Material that may be linked to, or accessed through, an article. The authors have granted ASM a non-exclusive, world-wide license to publish the Supplemental Material files. Please contact the corresponding author directly for reuse.
