# Supplementary material for: Metabolic changes associated with polysaccharide utilization reduce susceptibility to some β-lactams in Bacteroides thetaiotaomicron
Source: mSphere. 2024 Aug 7;9(8):e00103-24. doi: 10.1128/msphere.00103-24 (PMC11351048; doi:10.1128/msphere.00103-24)
Supplement: Supplemental figures, tables, methods — Supplemental figures, Tables S1 and S2, and methods. [file msphere.00103-24-s0001.docx]

**
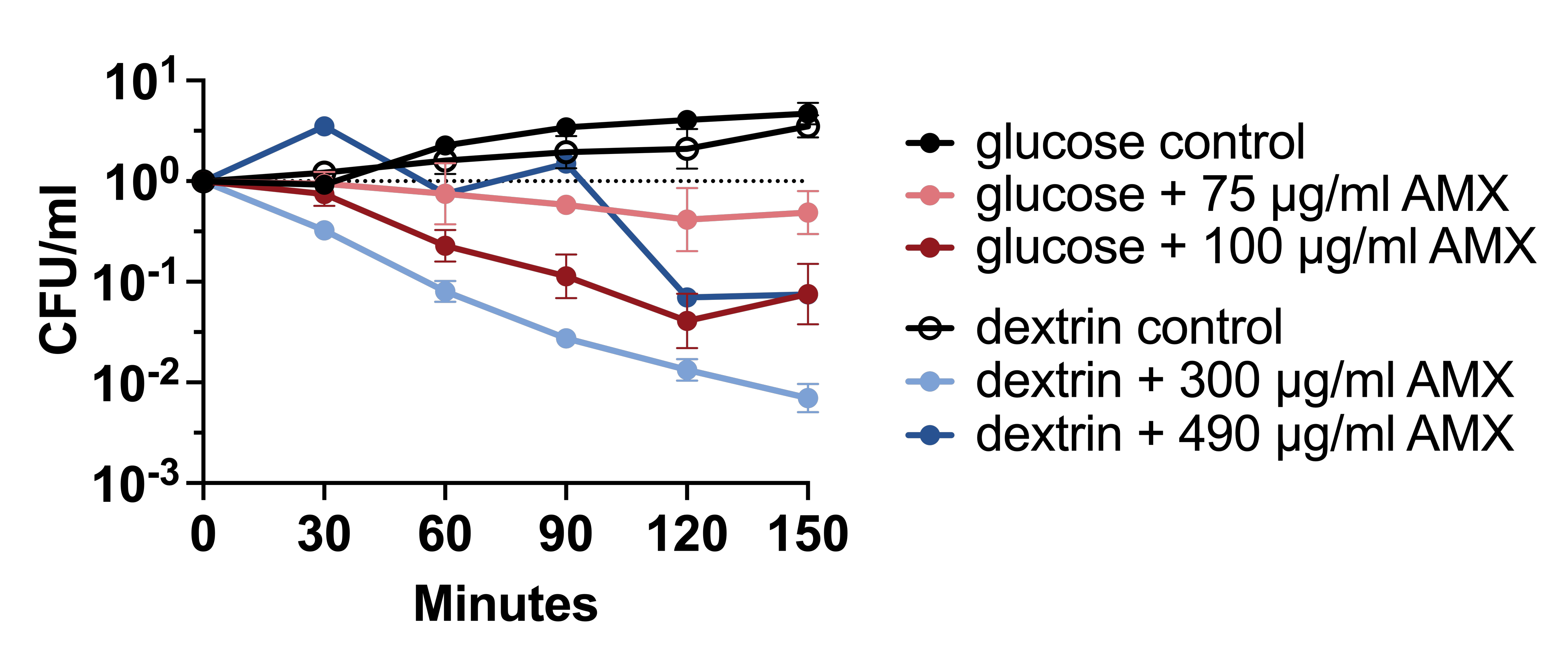
**

**Figure S1.** CFU/ml for *Bth* grown in MM-glucose or MM-dextrin and treated with the indicated AMX dose. Data are represented as the average change in CFU/ml compared to baseline ± SEM.

**
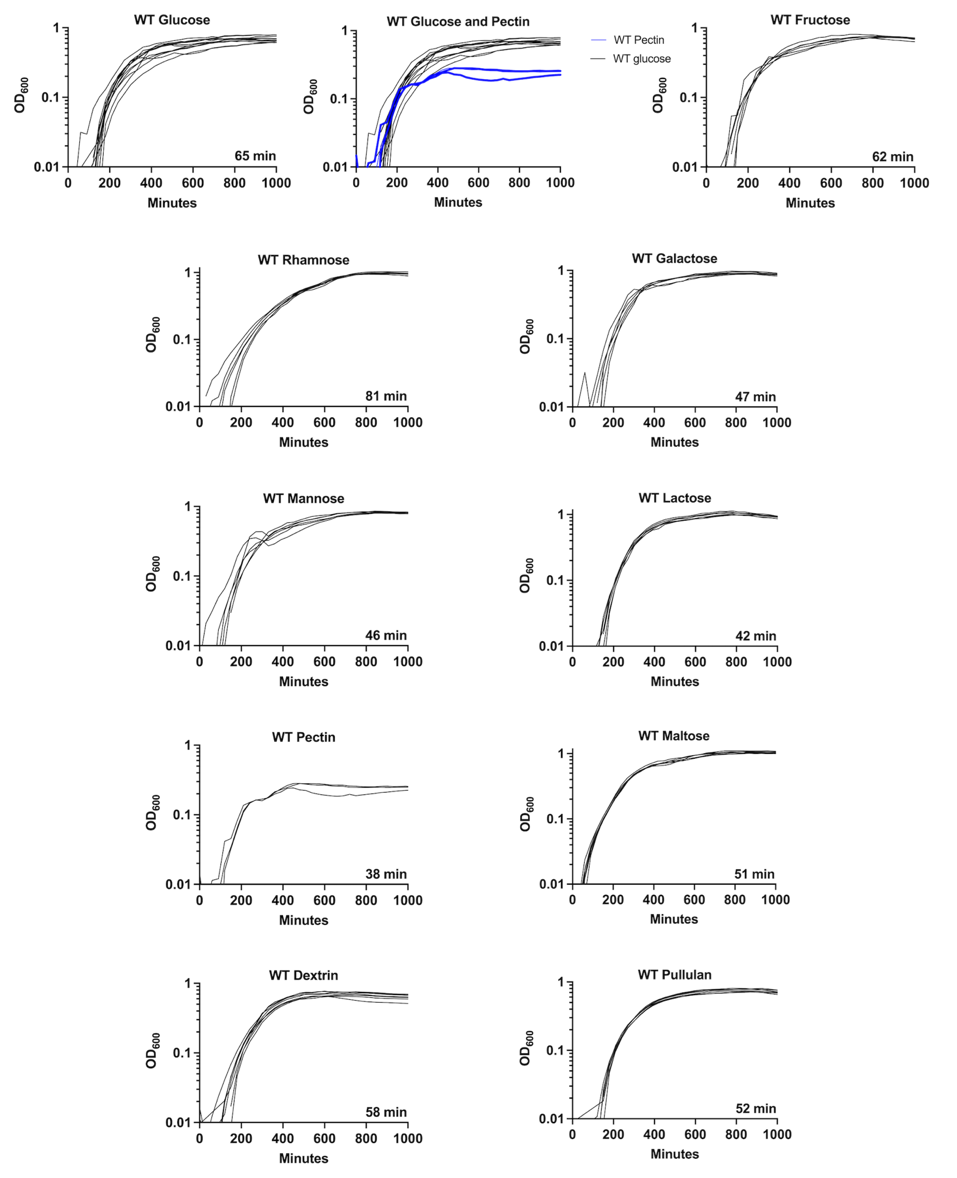
**

**Figure S2.** Growth curves and doubling times for WT *Bth* grown in MM with the indicated carbon source. Each line represents a biological replicate. All growth data can be found in Table S3.

**
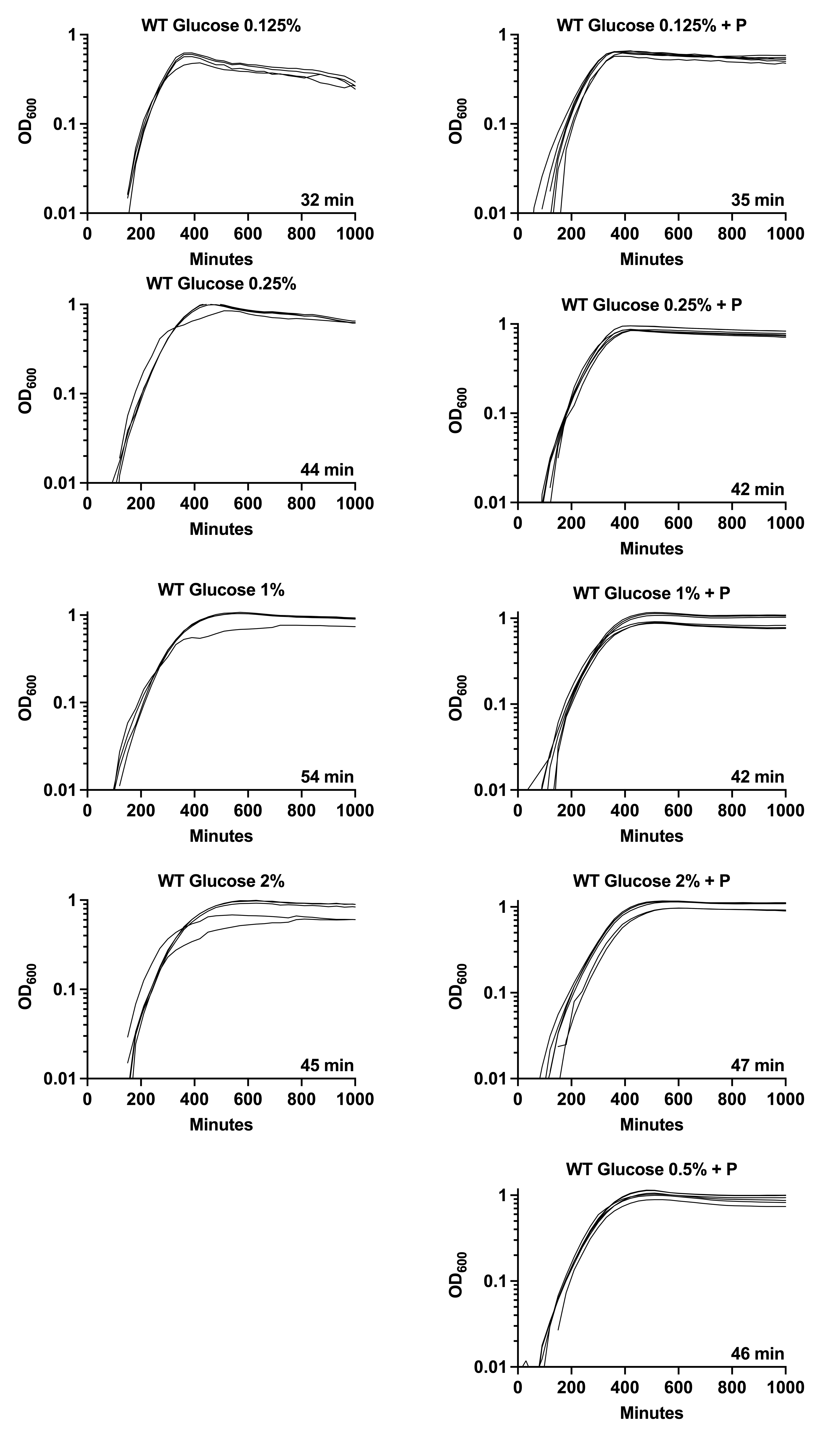
**

**Figure S3.** Growth curves and doubling times for WT *Bth* grown in MM with the indicated glucose concentration $\pm$ 0.5% pectin (P). Each line represents a biological replicate. All growth data can be found in Table S4.

**
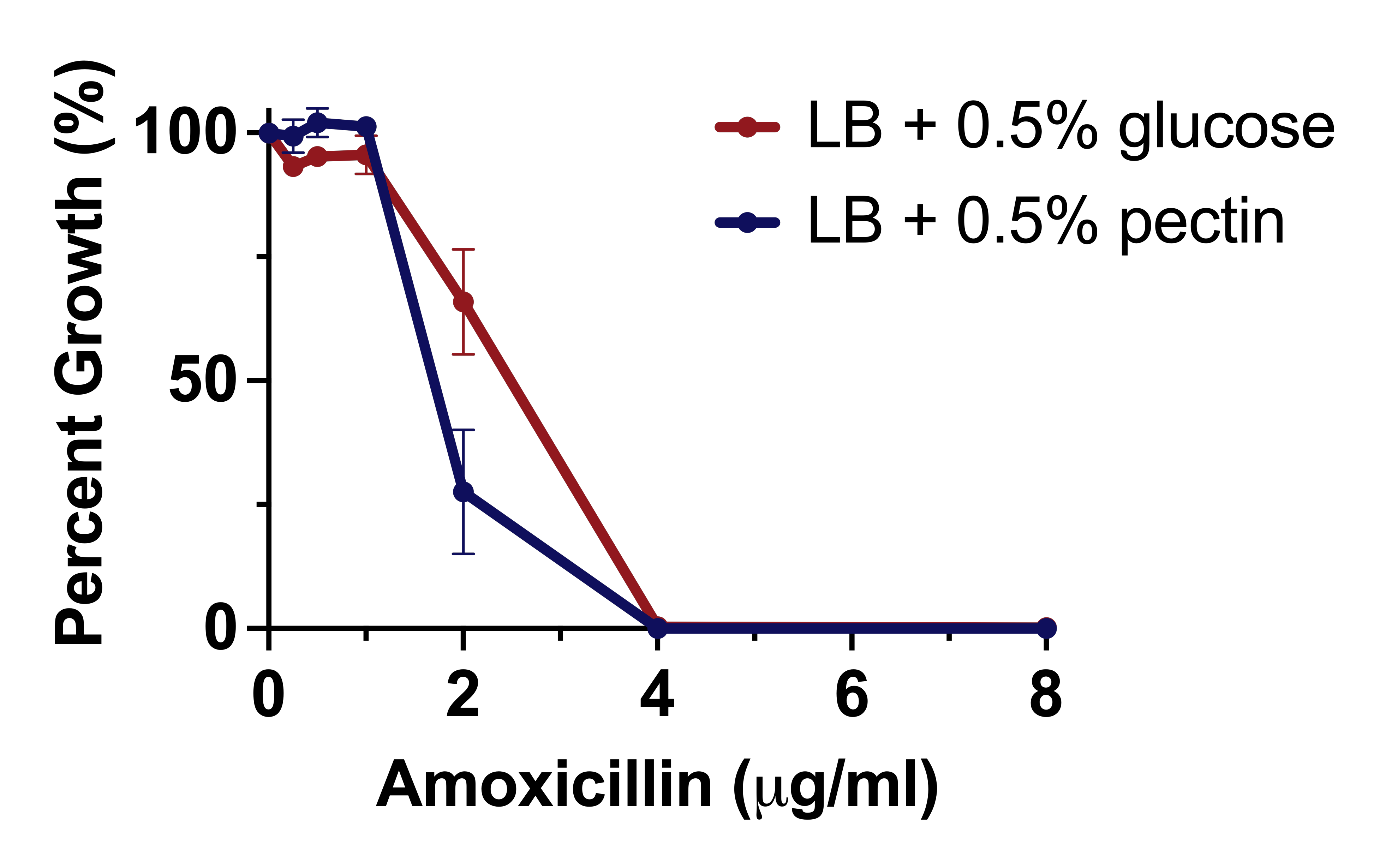
**

**Figure S4.** Percent growth plot for *E. coli* grown in LB supplemented with glucose or pectin and treated with AMX. Data are represented as the average percent growth compared with untreated control cultures ± SEM.

**
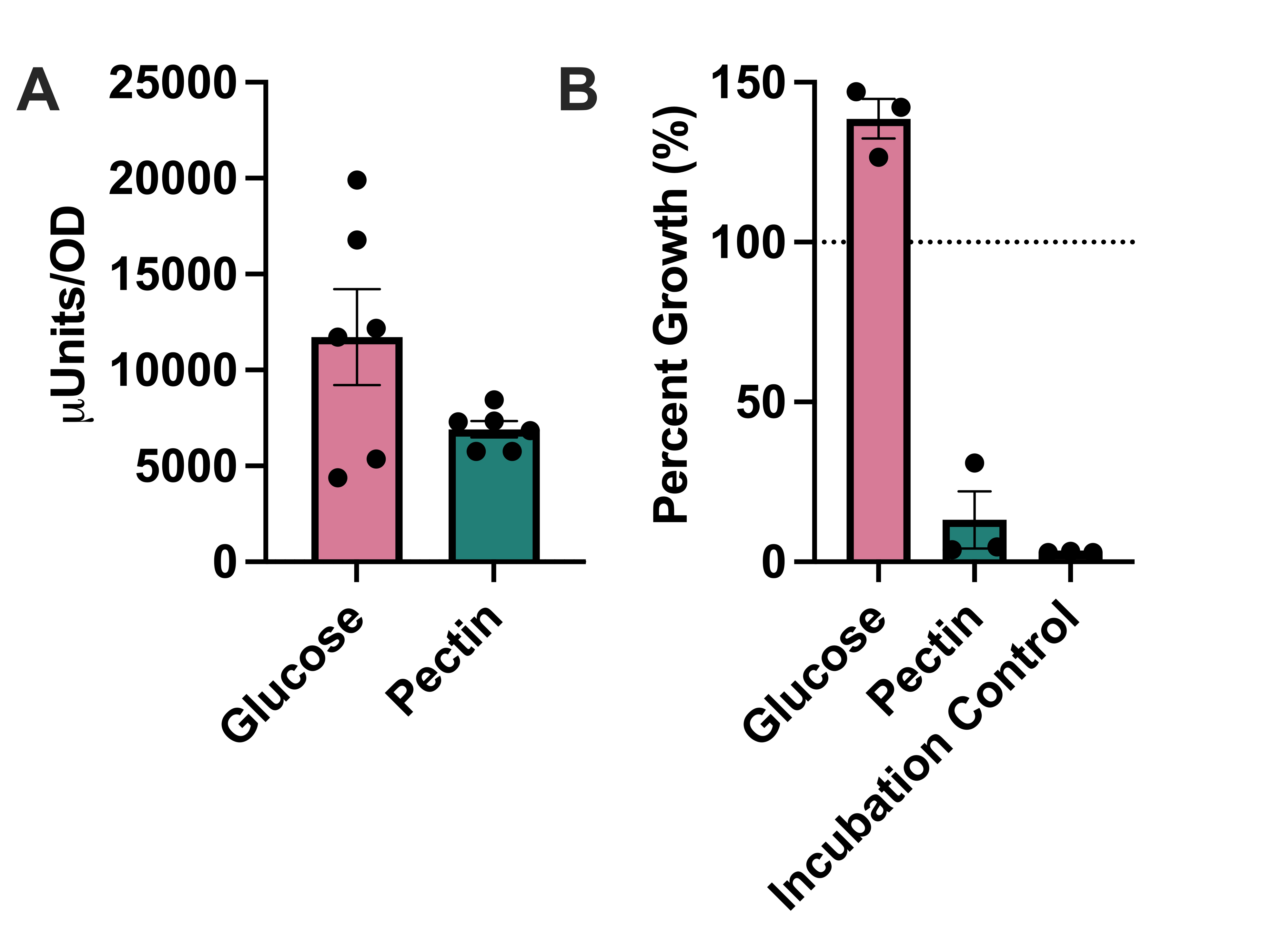
**

**Figure S5. (A)** $\beta$-lactamase levels in *Bth* grown in MM-glucose or MM-pectin. Data are represented as the average of six biological replicates ± SEM. One unit of $\beta$-lactamase is the amount of enzyme required to hydrolyze 1.0 μmole of nitrocefin per minute at pH 7.0 at 25°C. **(B)** Percent growth for *E. coli* exposed to 50 μg/ml AMX pre-incubated with *Bth* grown in MM-glucose or MM-pectin for 4 hours at 30°C. More growth indicates more $\beta$-lactamase activity. Data are represented as the average percent growth compared with untreated control cultures ± SEM.

**
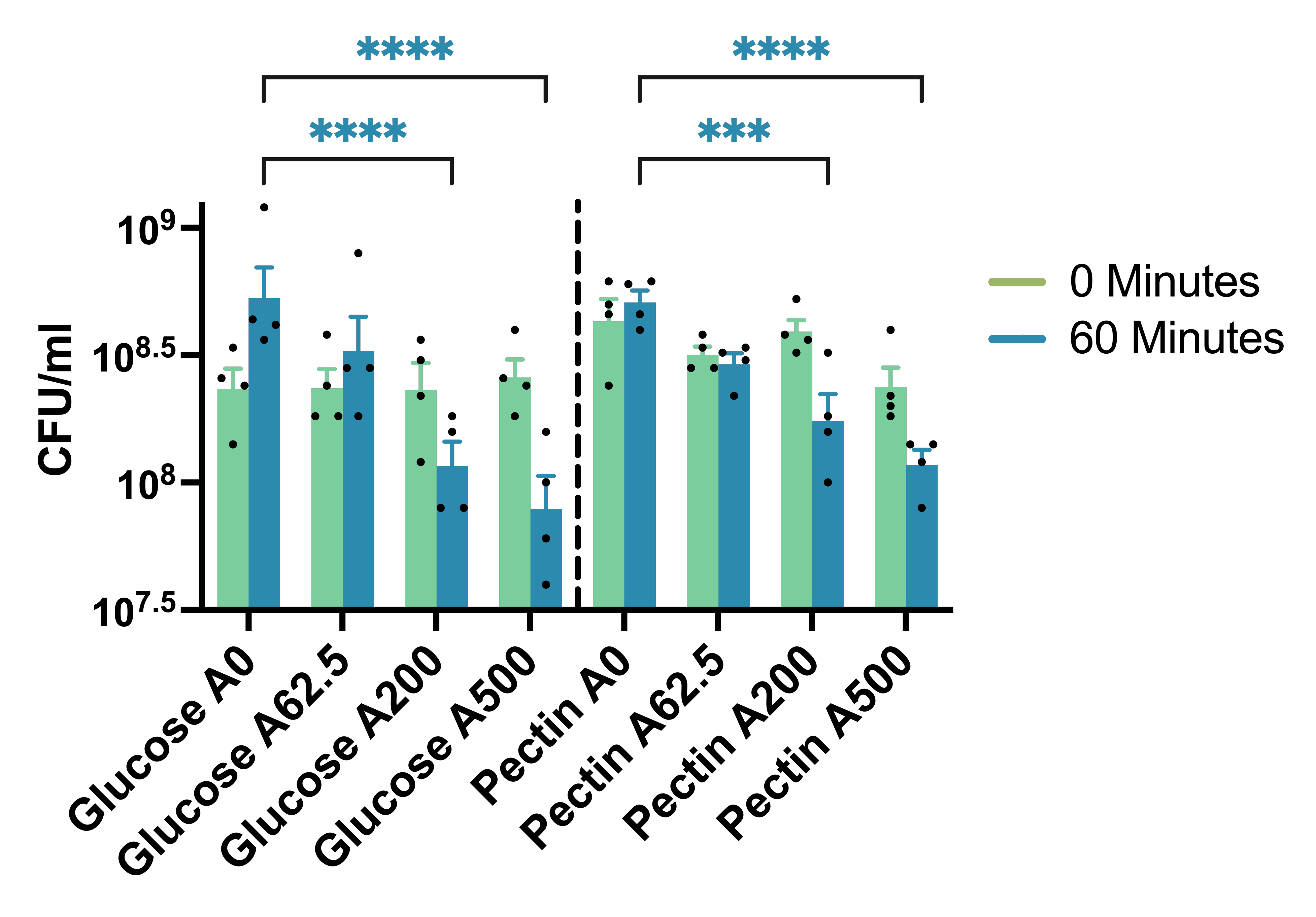
**

**Figure S6.** CFU/ml for the indicated conditions. Samples are paired so that each condition consists of four bacterial cultures sampled at 0 minutes and 60 minutes. ***p<0.001, ****p<0.0001; 2way ANOVA with Šidák correction for multiple hypothesis testing. A = amoxicillin dose (μg/ml).

**
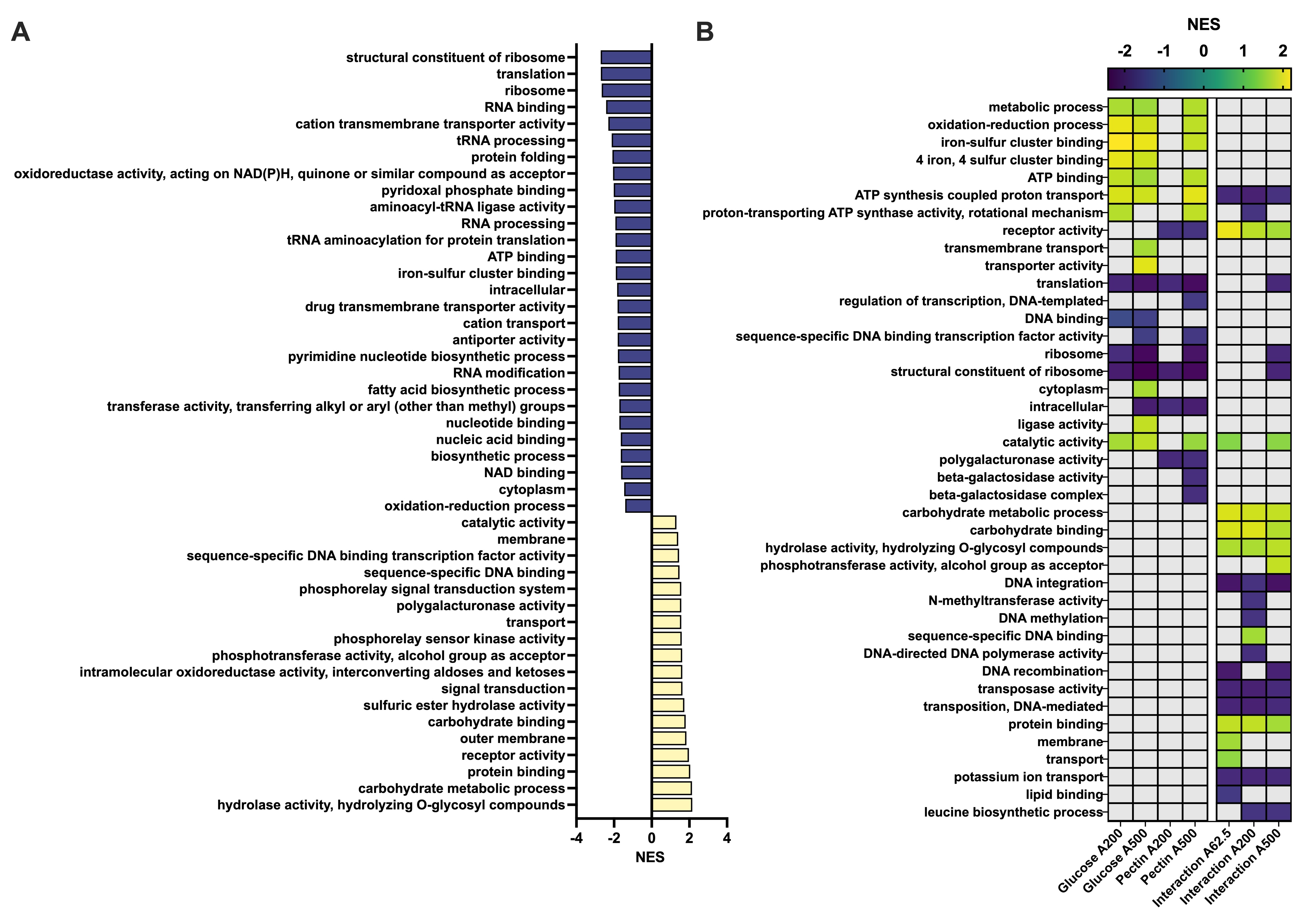
**

**Figure S7.** **(A and B)** Normalized enrichment scores (NES) for gene set enrichment analysis of GO pathways. Benjamini-Hochberg adjusted p-value < 0.05 and -1 ≤ log2foldchange ≤ 1. (A) Pectin T0 A0 samples vs glucose T0 A0 samples. (B) Two-way interactions between time and amoxicillin treatment at the indicated dose and three-way interactions between time, amoxicillin treatment, and carbon source at the indicated dose. Grey panels are non-significant. T = time (min), A = amoxicillin dose (μg/ml). For all conditions n=4.

**
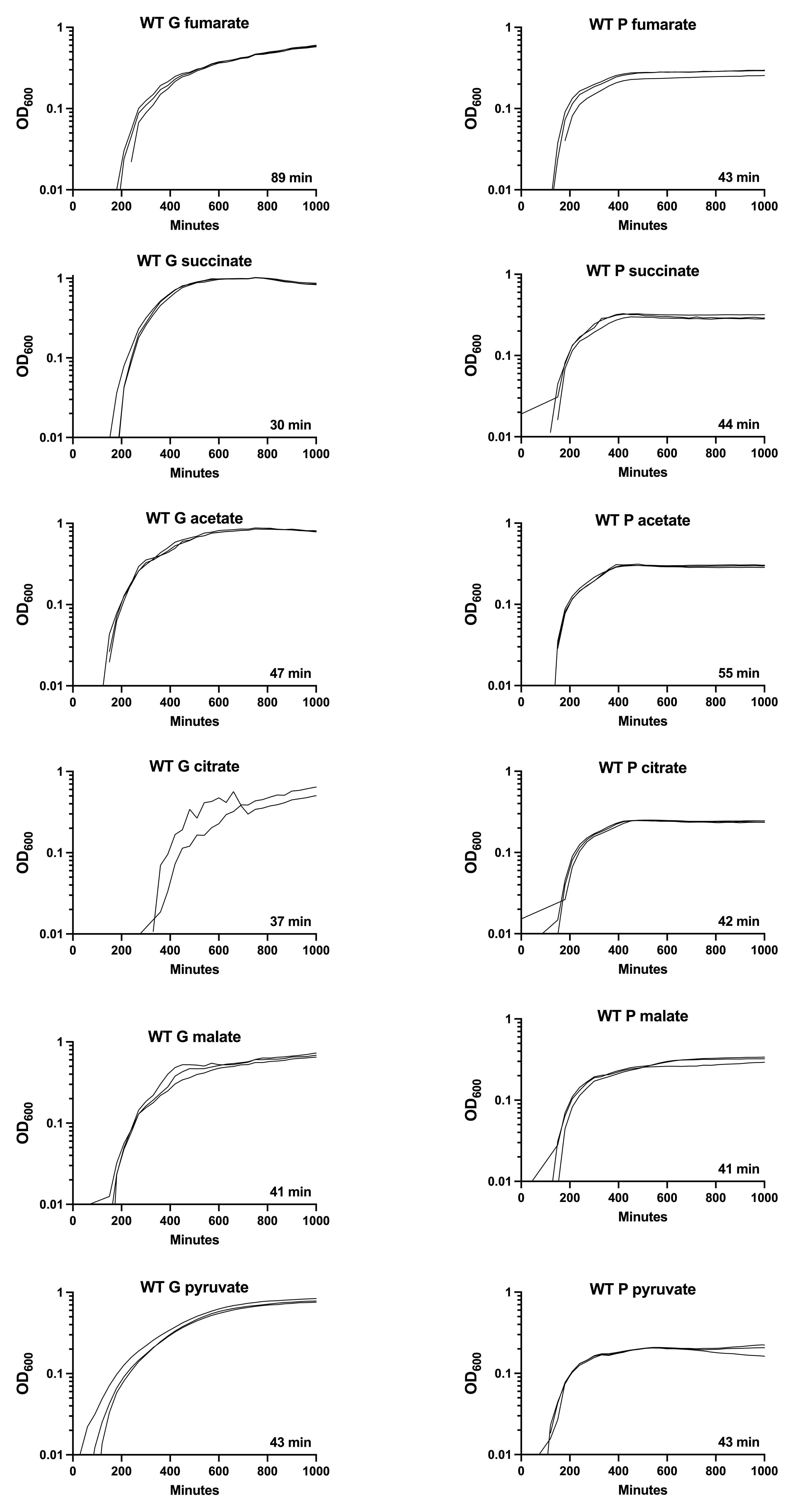
**

**Figure S8.** Growth curves and doubling times for WT *Bth* grown in MM with glucose (G) or pectin (P) and supplemented with the indicated metabolite. Each line represents a biological replicate. All growth data can be found in Table S5.

**
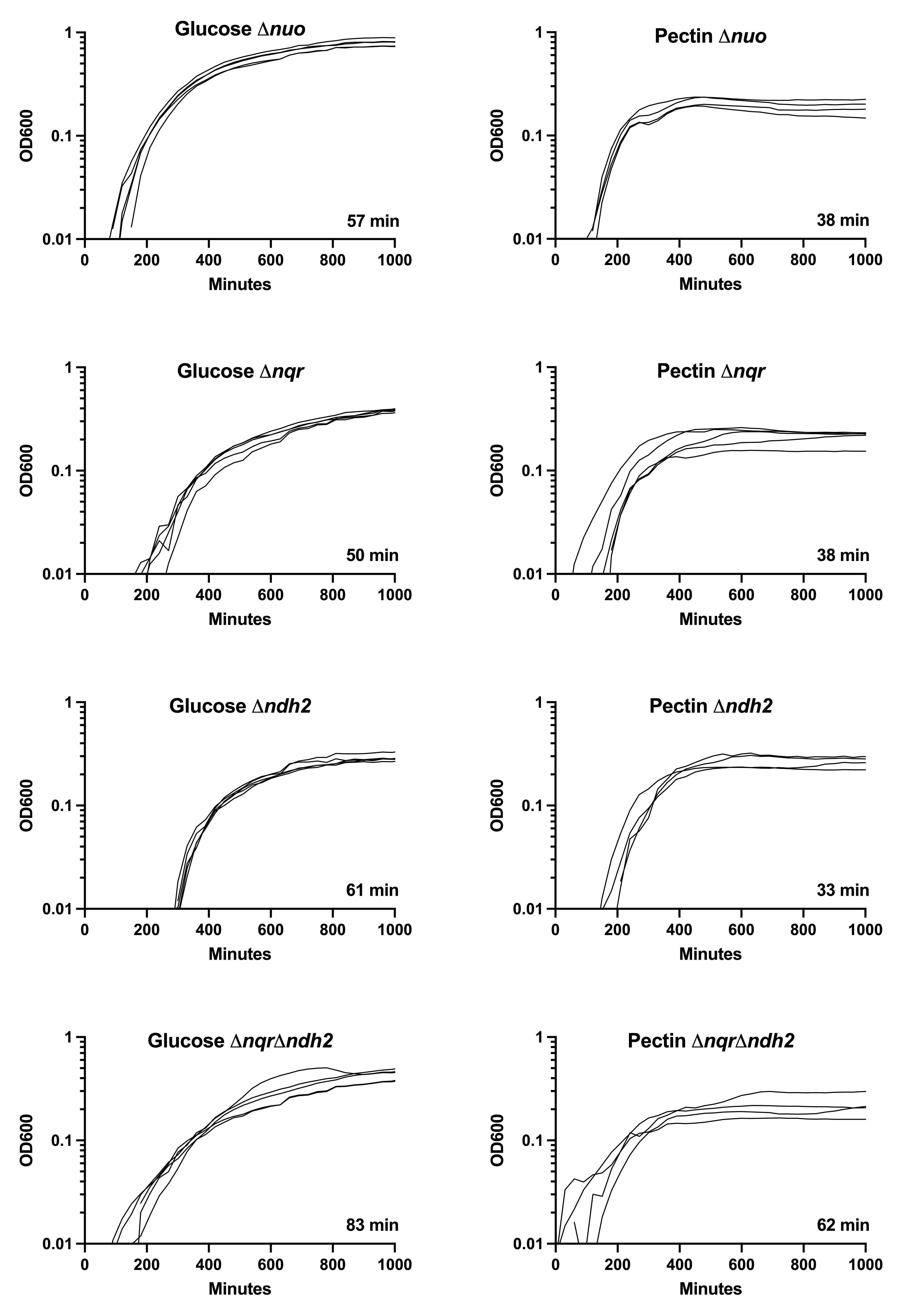
**

**Figure S9.** Growth curves and doubling times for *Bth* knockouts grown in MM-glucose or MM-pectin. Each line represents a biological replicate. All growth data can be found in Table S6.

**
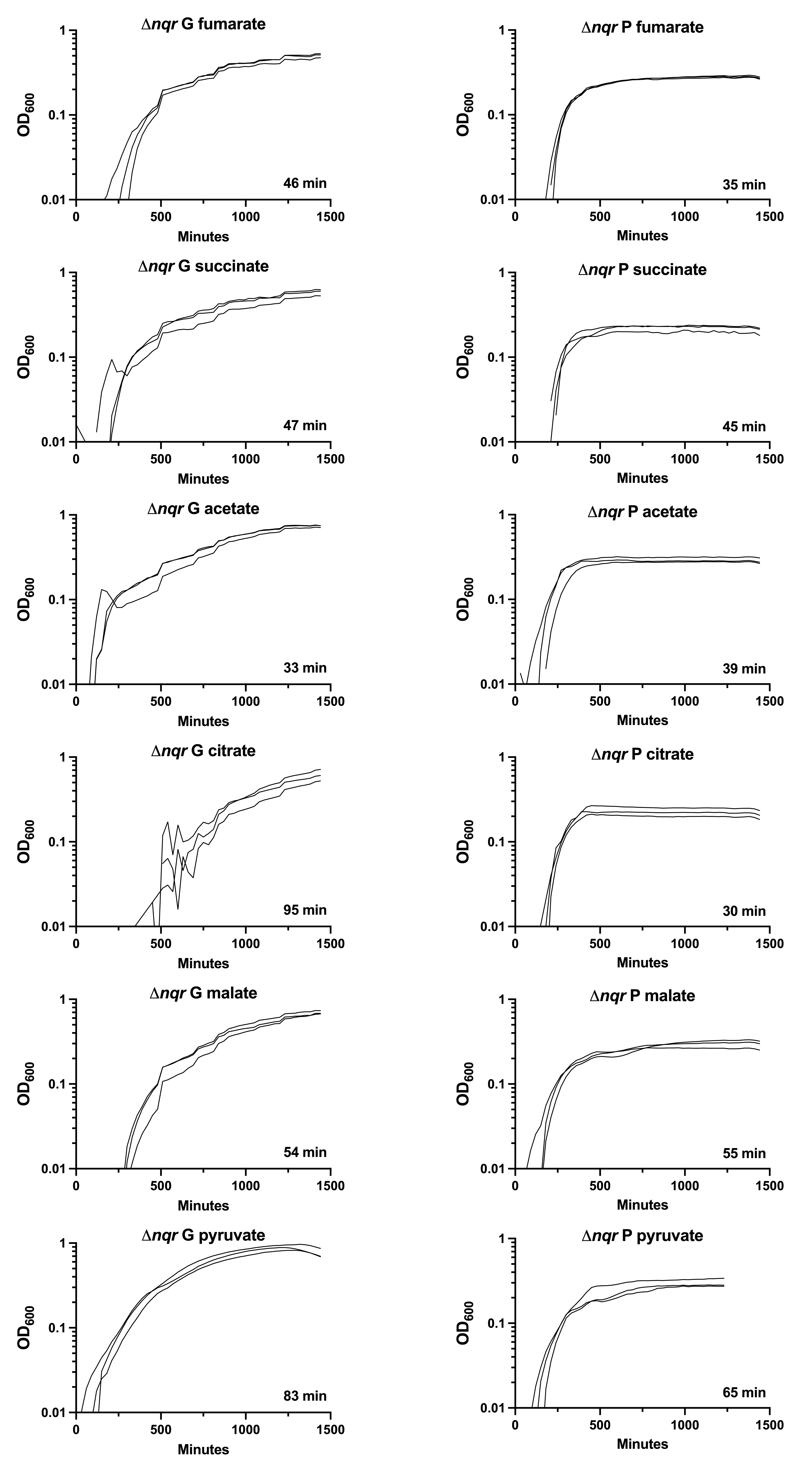
**

**Figure S10.** Growth curves and doubling times for *Bth* $\Delta$*nqr* grown in MM with glucose (G) or pectin (P) and supplemented with the indicated metabolite. Each line represents a biological replicate. All growth data can be found in Table S7.

**Table S1.** Strains and plasmids used in the present study.

| Strain | Description | Reference/Source |
| --- | --- | --- |
| ***Bacteroides thetaiotaomicron*VPI-5482** | Wild type | ATCC 29148 |
| ***Bacteroides thetaiotaomicron*** $\boldsymbol{\Delta}$***tdk*** | ATCC 29148 with *tdk* deletion; FUdR^R^; parent strain for deletions | (1) |
| ***Bacteroides thetaiotaomicron*** $\boldsymbol{\Delta}$***tdk***$\boldsymbol{\Delta}$***nuo*** | BT4058-4067 deletion | This study |
| ***Bacteroides thetaiotaomicron*** $\boldsymbol{\Delta}$***tdk***$\boldsymbol{\Delta}$***nqr*** | BT1155-1160 deletion | This study |
| ***Bacteroides thetaiotaomicron*** $\boldsymbol{\Delta}$***tdk***$\boldsymbol{\Delta}$***ndh2*** | BT0387 deletion | This study |
| ***Bacteroides thetaiotaomicron*** $\boldsymbol{\Delta}$***tdk***$\boldsymbol{\Delta}$***nqr***$\boldsymbol{\Delta}$***ndh2*** | BT1155-1160 deletion; BT0387 deletion | This study |
| ***Escherichia coli* MG1655** | Wild type | ATCC 700926 |
| ***Escherichia coli* S17 pir** | Maintenance strain for pExchange-*tdk* | (1) |
|  |  |  |
| Plasmid | Description | Reference/Source |
| **pExchange-*tdk*** | Derivative of pKNOCK-*bla-ermG*b carrying cloned *tdk* | (1) |
| **pExchange-*tdk*::*nuo*** | BT4058-4067 deletion | This study |
| **pExchange-*tdk*::*nqr*** | BT1155-1160 deletion | This study |
| **pExchange-*tdk*::*ndh2*** | BT0387 deletion | This study |

**Table S2.** Primers used in the present study.

| **Name** | **Sequence** |
| --- | --- |
| **BT4058 - BT4067 upstream FWD w/ SalI** | GCGGTCGACGTGGAAATCATCGGTATCAA |
| **BT4058 - BT4067 inside REV** | GAAACCGACTCTTTGGGG |
| **BT4058 - BT4067 inside FWD** | CCCCAAAGAGTCGGTTTCAATTTCAATAGCTGTTTAGATTGA |
| **BT4058 - BT4067 downstream REV w/ NotI** | GCGGCGGCCGCTTATTTGCATAAAGATTCAACAA |
| **BT4058 - BT4067 deletion screen FWD** | GAGGTCAGAAAAGGAAACCG |
| **BT4058 - BT4067 deletion screen REV** | ACATATCGTCCAAGGTCTTT |
| **BT1155 - BT1160 upstream FWD** | GCGGTCGACTTCAACCGGTTTTCCTGT |
| **BT1155 - BT1160 inside REV** | TCTTCGGAAGTTGATTTCCG |
| **BT1155 - BT1160 inside FWD** | CGGAAATCAACTTCCGAAGAGCTATATAAAATAATGGTTTCATA |
| **BT1155 - BT1160 downstream REV w/ NotI** | GCGGCGGCCGCTTTCTGAAGAAATACGGTGA |
| **BT1155 - BT1160 deletion screen FWD** | GAGTAGCTTGTGCACACGAT |
| **BT1155 - BT1160 deletion screen REV** | AAGTGGAAATGTTGAGCACC |
| **BT0387 upstream FWD w/ SalI** | GCGGTCGACAGAGTGCAAGAAAGAATTAG |
| **BT0387 inside REV** | TACTTTAACTTCTTGATAATATTTACC |
| **BT0387 inside FWD** | GGTAAATATTATCAAGAAGTTAAAGTAAATAGTCATACGATTGGTATTT |
| **BT0387 downstream REV w/ NotI** | GCGGCGGCCGCTTCACTTTTGCAATCTACAT |
| **BT0387 deletion screen FWD** | CGTTTTTGGAGCATCATTAG |
| **BT0387 deletion screen REV** | CCAATTTGGACACGATTTCC |

**Table S3.** OD measurements and calculations for doubling times for wildtype *Bth* grown in minimal media containing different carbon sources. Provided as an Excel file.

**Table S4.** OD measurements and calculations for doubling times for wildtype *Bth* grown in minimal media containing different concentrations of glucose. Provided as an Excel file.

**Table S5.** OD measurements and calculations for doubling times for wildtype *Bth* grown in minimal media containing either glucose or pectin and supplemented with different metabolites. Provided as an Excel file.

**Table S6.** OD measurements and calculations for doubling times for *Bth* knock out strains grown in minimal media containing either glucose or pectin. Provided as an Excel file.

**Table S7.** OD measurements and calculations for doubling times for *Bth* $\Delta$*nqr* in minimal media containing either glucose or pectin and supplemented with different metabolites. Provided as an Excel file.

**SUPPLEMENTAL MATERIALS AND METHODS**

***Bth* kill curve.** Overnight *Bth* cultures were diluted to 0.05 OD_600_ in MM-glucose or MM-dextrin and grown to 0.1 OD_600_. Next, amoxicillin was added to MM-glucose cultures at 75 $\mu$g/ml or 100 $\mu$g/ml and to MM-dextrin cultures at 300 $\mu$g/ml or 490 $\mu$g/ml. Cultures were incubated anaerobically at 37°C and samples were taken for CFU plating at 0 (before addition of amoxicillin), 30, 60, 90, 120, and 150 minutes. For CFU determination, cells were serially diluted 10-fold in PBS and plated on mGAM. Plates were incubated anaerobically at 37°C until colonies were visible for counting (~20 h).

$\boldsymbol{\beta}$**-lactamase enzyme assay.** Overnight *Bth* cultures were diluted to 0.05 OD_600_ in MM-glucose or MM-pectin and grown for ~5 hours. $\beta$-lactamase activity was measured using the Beta-Lactamase Activity Assay Kit (Sigma-Aldrich) following the manufacturer’s instructions. Absorbance was measured on a SpectraMax M3 microplate reader (Molecular Devices LLC). Absorbance measurements were normalized to cell culture OD_600_ readings.

***E. coli* MIC determination using AMX pre-incubated with *Bth*.** First, *Bth* overnight cultures were diluted to 0.05 OD_600_ in MM-glucose or MM-pectin and grown until ~0.1 OD_600_. Next, cultures were treated with 100 $\mu$g/ml amoxicillin. After four hours of anaerobic incubation at 30°C, the supernatant was collected and filtered. Overnight cultures of *E. coli* were diluted 10,000-fold in LB. Using the broth dilution method, filtered supernatant was added to *E. coli* at 50 $\mu$g/ml, then serially diluted two-fold (2). An incubation control was performed, which used 100 $\mu$g/ml amoxicillin that had been incubated for four hours at 30°C in cell-free media and added to *E. coli* at 50 $\mu$g/ml*. E. coli* was incubated aerobically at 37°C for ~20 h, after which OD_600_ readings were taken on a SpectraMax M3 microplate reader (Molecular Devices LLC, San Jose, CA, USA).

**SUPPLEMENTAL REFERENCES**

1. Koropatkin NM, Martens EC, Gordon JI, Smith TJ. 2008. Starch catabolism by a prominent human gut symbiont is directed by the recognition of amylose helices. Structure 16:1105-1115.

2. Wiegand I, Hilpert K, Hancock REW. 2008. Agar and broth dilution methods to determine the minimal inhibitory concentration (MIC) of antimicrobial substances. Nature Protocols 3:163-175.
